# Tailoring the metal electrode morphology via electrochemical protocol optimization for long-lasting aqueous zinc batteries

**DOI:** 10.1038/s41467-022-31461-7

**Published:** 2022-06-27

**Authors:** Qing Li, Ao Chen, Donghong Wang, Yuwei Zhao, Xiaoqi Wang, Xu Jin, Bo Xiong, Chunyi Zhi

**Affiliations:** 1 Department of Materials Science and Engineering, City University of Hong Kong, 83 Tat Chee Avenue, Kowloon, Hong Kong 999077 China; 2 Hong Kong Center for Cerebro-Cardiovascular Health Engineering (COCHE), Shatin, NT, HKSAR China; 3 Research Institute of Petroleum Exploration & Development (RIPED), PetroChina, Research Center of New Energy, No. 20 Xueyuan Road Haidian District, Beijing, 100083 China

**Keywords:** Energy storage, Batteries, Engineering, Materials for energy and catalysis, Electrochemistry

## Abstract

Aqueous zinc metal batteries are a viable candidate for cost-effective energy storage. However, the cycle life of the cell is adversely affected by the morphological evolution of the metal electrode surface upon prolonged cycling. Here, we investigate different electrochemical protocols to favour the formation of stable zinc metal electrode surface morphologies. By coupling electrochemical and optical microscopy measurements, we demonstrate that an initial zinc deposition on the metal electrode allows homogeneous stripping and plating processes during prolonged cycling in symmetric Zn||Zn cell. Interestingly, when an initially plated zinc metal electrode is tested in combination with a manganese dioxide-based positive electrode and a two molar zinc sulfate aqueous electrolyte solution in coin cell configuration, a specific discharge capacity of about 90 mAh g^−1^ can be delivered after 2000 cycles at around 5.6 mA cm^−2^ and 25 °C.

## Abstract (web summary)

Long-lasting zinc metal electrodes are crucial in developing commercial zinc-based batteries. Here, the authors investigate the different morphology evolution between the stripping and plating process and propose electrochemical protocols to prolong the lifespan of zinc anodes.

## Introduction

The growing need for energy necessitates the development of various energy storage technologies beyond lithium-ion batteries (LIBs)^1–3^, and aqueous rechargeable Zn-based batteries (RZBs) are deemed one of the most promising prospective candidates for the next generation of large-format energy storage technology with cost efficiency, intrinsic safety, low toxicity, and acceptable energy density^4–7^. To date, the Zn metal anode (ZMA) has been recognized as an ideal anode for RZBs, offering a high theoretical capacity of 812 mAh g^−1^ and a competitive electrochemical potential of −0.76 V vs. the standard hydrogen electrode (SHE)^8^. Nevertheless, the dendrite issue of ZMA restricts the anode lifespan, which has become a bottleneck in the practical application of RZBs^9–15^. Considerable effort has been made to delve into feasible strategies for dendrite suppression through electrode design^16,17^, interface modification^8,18^, and electrolyte optimization^19,20^. However, the focus has been concentrated on the plating process, while the stripping process of the ZMA is not generally considered.

A representative model to elucidate the plating/stripping behaviour is the symmetric cell, which offers the possibility to investigate the electrochemical behaviour of the ZMA and avoids the disturbance of the cathode side. As such, symmetric cells have been extensively applied in related research. The current analysis focuses on the cycling time before the cell short circuit. Interestingly, the symmetric cell voltage profile is not entirely flat. Additionally, the shape and variation of the voltage can provide more detailed information^21^. However, understanding of the voltage profile for the Zn||Zn symmetric cell system has been insufficient to date.

In addition, the uneven plating and stripping, as well as the side effects during battery operation, render the ZMA an irreversible electrode, implying that the symmetric cell could easily become asymmetric during cycling^21–24^. The symmetric cell consists of two electrodes: the initial stripped Zn electrode (S-Zn) and the initial plated Zn electrode (P-Zn), which show different electrochemical behaviours. In the research on Li metal anode, the asymmetric behaviour of Li||Li symmetric cells was first investigated by Lee and coworkers^25^. They found that the initially plated electrode is more reversible than the stripped electrode. Zhang et al. validated the asymmetric behaviour in full cells with a Li-containing cathode (LiFePO_4_) or the Li-free cathode (FePO_4_), and a Li anode initially plated Li anode demonstrated a prolonged lifespan when coupled with the LiFePO_4_ cathode^21^. The different behaviours of the initially stripped and plated electrodes can be instructive for developing more rational asymmetric cycling protocols. Recent works on ZMA optimization mainly focus on the plating process and morphology, while indeed, the stripping behaviour should be considered to be equally important^26–28^. In particular, unlike the typical cathode materials of LIBs with an initially discharged state, cathode materials of RZBs (MnO_2_, V_2_O_5,_ etc.) are in a charged state^29^. That is, for RZBs, the ZMA will first encounter a stripping process that distinguishes it from the Li anode. The comprehension of the stripping process, which is even more essential in practice to construct a robust ZMA, has long been neglected in research on the ZMA^30^.

**Fig. 1:**
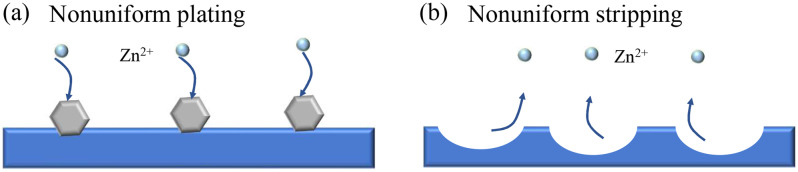
Schematic illustrating the difference between plating and stripping. **a** Schematic of the nonuniform plating process. **b** Schematic of the nonuniform stripping process. The pale blue spheres represent Zn ions, the solid blue substrate represents the Zn metal electrode, and the grey hexagons represent the deposited Zn lamellae. Different morphologies can be obtained due to the different initial processes (stripping or plating).

The common research scenario is based on Fig. 1a with a flat Zn foil initially plated. The nonuniform nucleation formed in the initial cycles results in dendrite accumulation after repeated cycles. However, the practical situation is the opposite. The initial process in RZBs is stripping instead of plating, and pits (i.e., formation of inhomogeneous void spaces caused by nonuniform metal dissolution) will first form on the Zn surface (Fig. 1b). The difference between Fig. 1a and 1b exemplifies the asymmetry for P-Zn and S-Zn, serving as a reminder of the importance of investigating the stripping behaviour of ZMA.

Herein, to deeply understand both stripping/plating behaviour of the ZMA, the time-voltage profiles of two individual electrodes (P-Zn and S-Zn in the same symmetric cell) are decoupled and monitored separately. The morphology evolution is further linked with the voltage response, providing insights into the voltage–time curves. Additionally, the different morphologies and performance evolutions of P-Zn and S-Zn are articulated. The dendrites grew in the pits of S-Zn, while dendrites grew on the protrusion of P-Zn. The dendrites formed from the pits are more heterogenous than those on the surface. Through observation, a “predeposition” strategy is proposed to alleviate dendrite formation. With a uniform predeposition treatment of the ZMA surface, a uniform stripping/plating process was obtained in the following cycles for the predeposited Zn anode (PD-Zn) due to the interaction with the initially deposited layer. The symmetric PD-Zn||PD-Zn cell show a prolonged life (over 1000 h), and the full PD-Zn||MnO_2_ cell is cycled for 2000 cycles without a short circuit.

## Results

### Electrochemical and microscopy investigation of the symmetric Zn||Zn cells

A typical voltage profile of a Zn||Zn symmetric cell is shown in Supplementary Fig. 1. The voltage profiles during plating and stripping are symmetric despite some changes in voltage shape as the cycles progress. This configuration can be misleading since the two electrodes are considered to be identical. To further distinguish the behaviour of each electrode, a three-electrode configuration illustrated in Supplementary Fig. 2 is applied^31^, in which the working electrode and the counter electrode include two Zn foils responsible for stripping/plating, and the reference electrode is a separate Zn electrode. The separate voltage profiles of the working electrode and counter electrode are monitored by potentiostat channels, and the overall voltage between the working electrode and counter electrode is also recorded. The process is monitored in situ by an optical microscope to investigate the relationship between the voltage profile and morphology evolution.

**Fig. 2:**
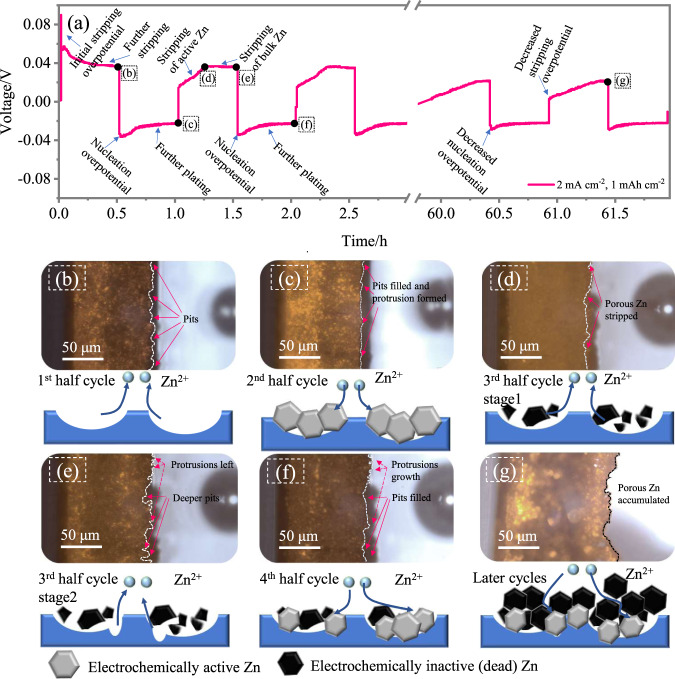
Potential profiles and morphology evolution of the initially stripped Zn metal electrodes. **a** Voltage evolution of S-Zn. **b**–**g** Optical micrographs and the corresponding schematic figures depicting the morphology of the positions marked in (**a**).

Figure 2a shows the voltage evolution of S-Zn during the stripping and plating processes. In the beginning, the abrupt voltage increase to approximately 0.09 V corresponds to the resistance of the initial stripping from the uncycled Zn foil. At the end of the initial stripping, pits can be observed (arrows), implying that Zn dissolution is not uniform (Fig. 2b). Instead, Zn is prone to strip from positions that have already been activated by previous dissolution, thus forming pits. The plating voltage of the first plating process then gradually increases with time, indicating that the beginning of plating requires a higher overpotential activation, and the consequent Zn plating on the deposited Zn is relatively easy. The morphology of Zn shown in Fig. 2c in the 2nd half cycle indicates that the pit is filled with deposited Zn with protrusions appearing (arrows), suggesting that Zn prefers to deposit in the pit area. In the subsequent stripping process (Fig. 2a), the stripping voltage starts from 0.02 V vs. Zn/Zn^2+^ and then increases to 0.04 V vs. Zn/Zn^2+^ (Fig. 2a–d) in the middle and remains at this level until the end of stripping (Fig. 2a–e). The voltage profile for this second stripping process can be divided into two parts: the rising voltage (positions c, d in Fig. 2a) and the stable voltage (positions d, e in Fig. 2a). The initial low voltage is attributed to the preexisting porous Zn deposited in the former process, which is easy to strip. Then, the voltage gradually rises during the removal of the porous Zn layer. When the porous Zn has been depleted, the stripping process is then switched to bulk Zn (i.e., uncycled Zn of the remaining Zn foil), which maintains a similar higher level of voltage. In this process, deeper pits up to 14 μm are formed at the positions of Fig. 2a–e compared with those of Fig. 2a–d, indicating that this process of stripping and plating for a single electrode is not highly reversible and that each cycle can consume some extra bulk Zn. In the following plating process, the pits are filled with deposited Zn (Fig. 2f). Several cycles later, the Zn electrode gradually evolves into a highly heterogeneous and dendritic morphology (Fig. 2g), and the stripping voltage in the later cycles of Fig. 2a gradually increases with time and is relatively lower than that of the preliminary cycles. The reason for this is that the porous, mossy Zn layer is formed during constant stripping and plating. Some of the mossy Zn can break down and form “dead Zn” (i.e., Zn metal regions that are electronically disconnected from the electrode current collector), which can be observed at the bottom of the glass cell shown in Supplementary Fig. 3. “Dead Zn” is one of the culprits of the poor reversibility of the Zn anode (Supplementary Fig. 4).

**Fig. 3:**
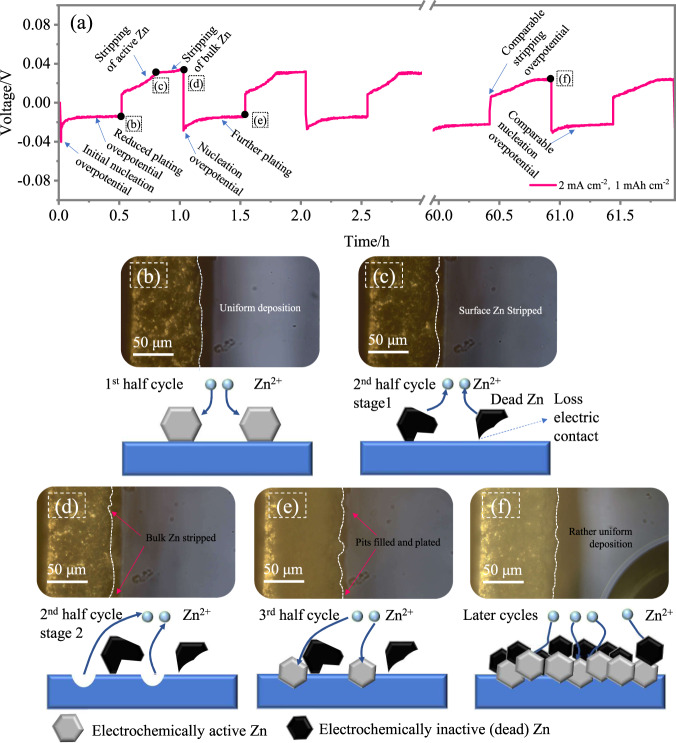
Potential profiles and morphology evolution of the initially plated Zn metal electrodes. **a** Voltage evolution of P-Zn. **b**–**f** The optical micrographs and the corresponding schematic figures displaying the morphology of the positions marked in (**a**).

In the case of P-Zn, as shown in Fig. 3a, *t* the voltage first decreases to −0.04 V vs. Zn/Zn^2+^ and quickly returns to a stable value of approximately −0.02 V vs. Zn/Zn2+ (Fig. 3a, b). This initial decrease is attributed to. This initial increase is attributed to nucleation on the fresh Zn surface requiring more energy and the fact that plating on the deposited Zn is much easier. The absolute value of the plating voltage was only half of the voltage value of ∼0.09 V vs. Zn/Zn^2+^ needed to activate the stripping process (Fig. 2a), indicating that stripping from uncycled Zn was more difficult than plating on the Zn surface. As shown in Fig. 3b, Zn is deposited onto the Zn surface, and the deposited layer is observed. The next stripping voltage profile (Fig. 3a) exhibits a similar curve shape, as shown in Fig. 2a, with two stages: the voltage increase stage (porous Zn) and voltage maintenance stage (bulk Zn). This process can be validated by the morphology evolution shown in Fig. 3c, d. The stripping starts with the porous Zn layer (Fig. 3c), and then stripping of bulk Zn leads to the formation of small pits (approximately 5 μm) in the bulk (Fig. 3d with arrows indicating the pit area), which is consistent with the stripping process in Fig. 2c. In the subsequent plating process, an extra Zn layer is deposited with decreasing voltage, as shown in Fig. 3e (Fig. 3a–e). The voltage profile for this electrode after several cycles is similar to that of S-Zn (later cycles in Fig. 2a) due to the formation of a porous and mossy Zn layer on the surface. Additionally, the morphology for the P-Zn electrode is relatively uniform and compact after several cycles compared to S-Zn (Fig. 3f vs. Fig. 2g).

Some similarities between P-Zn and S-Zn are observed: the voltage curves of the stripping and plating processes are similar. All stripping processes can be divided into two parts: porous Zn (i.e., deposited from the previous cycles) and bulk Zn. They reveal different kinetics: the dissolution is kinetically faster for porous Zn, resulting in a low stripping voltage in the initial half stripping process. In contrast, the dissolution of bulk Zn requires higher voltage activation in the latter half stripping process, indicating slower kinetics. The overpotential for the plating process is constantly decreasing for both P-Zn and S-Zn, and the reason is that the consequent plating process on the porous Zn is more favourable than the plating on the fresh Zn. The different voltage responses of the stripping and plating processes prove that stripping and plating are not fully reversible. Additionally, the voltage evolution can reflect different stages of stripping and plating.

The different morphological evolution of S-Zn and P-Zn shows that the symmetric cells become asymmetric. It also highlights the importance of the initial cycles, which encounter critical morphology changes to form a reconstructed interface layer and will further trigger the consequent morphology evolution. Similarly, for Zn powder-based electrodes (i.e., electrodes produced by slurry casting of Zn on a Cu substrate), the overpotential of the S-Zn powder electrode gradually increases due to contact loss between the Zn particles (Supplementary Fig. 5a). In contrast, the P-Zn powder electrode shows a stable overpotential during 20 h of cycling (Supplementary Fig. 5b, c).

### Ex situ microscopy investigation of the cycled Zn metal electrodes

**Fig. 4:**
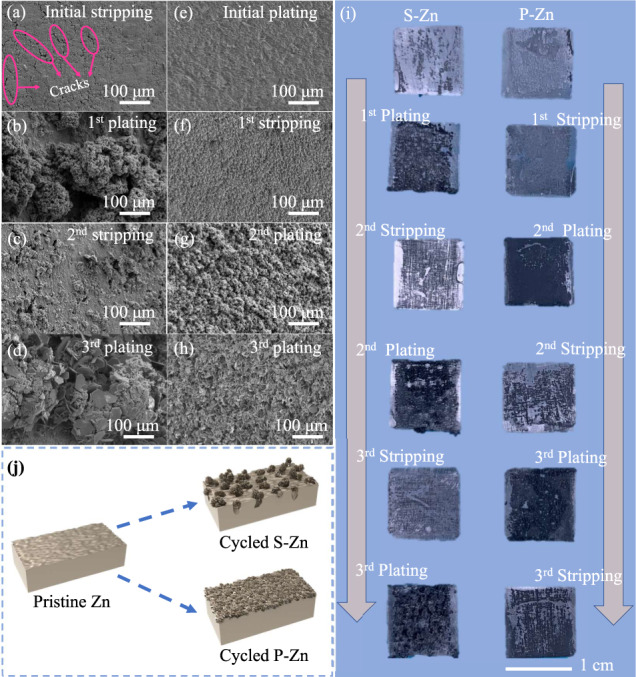
Ex situ microscopy investigation of Zn metal electrodes after stripping or plating. **a**–**d** Side view SEM images of the Zn metal electrode after the initial stripping, the following 1st plating, 2nd stripping, and the 3rd plating at an angle of 45°; **e**–**h** side view SEM images of the Zn metal electrode after initial plating, the following 1st stripping, 2nd plating, and the 3rd stripping; **i** photographic picture of the disassembled Zn metal electrode; **j** schematic of the morphology evolution of cycled S-Zn and P-Zn.

To ascertain the difference between S-Zn and P-Zn, scanning electron microscopy (SEM) measurements were carried out for detailed observations. Different states of the Zn electrode were obtained at a current density of 10 mA cm^−2^ for 0.5 h for each stripping and plating segment. For the S-Zn electrode, “cracks” (i.e., preferential Zn dissolutions along the thickness direction) is observed for the initial stripping (side view at an angle of 45° in Fig. 4a, cross view in Supplementary Fig. 6a), indicating nonuniform stripping. The Zn dendrite cluster that forms in the following plating (side view at an angle of 45° in Fig. 4b, cross view in Supplementary Fig. 6b) implies that Zn was deposited prior to the cracks, confirming the observation in Fig. 2. Further Zn metal dissolution enlarges the cracks formed in the initial stripping (side view at an angle of 45° in Fig. 4c, cross view in Supplementary Fig. 6c), validating the irreversibility, and each stripping process requires extra Zn stripping from the bulk Zn. After that, a larger dendrite cluster with a width surpassing 200 μm appears in the next plating (side view at an angle of 45° in Fig. 4d, cross view in Supplementary Fig. 6d). In contrast, P-Zn demonstrates a different dendrite growth route, and the morphology of the initial Zn deposition is compact and dense lamellar structures (Fig. 4e and Supplementary Fig. 6e). This lamellar structure transitions to uniform and porous Zn in the following cycles (Fig. 4f and Supplementary Fig. 6f). The Zn deposits in the next plating (2nd plating) are uniformly mossy and porous Zn (Fig. 4g and Supplementary Fig. 6g). After the 3rd plating, the P-Zn electrode is more uniform (Fig. 4h and Supplementary Fig. 6h) than the S-Zn electrode (Fig. 4d and Supplementary Fig. 6d). The ex situ photographs (Fig. 4i) and images obtained from the optical microscope (Supplementary Figs. 7 and 8) also support the different behaviours of S-Zn and P-Zn (the morphology of the P-Zn electrode is more uniform in the latter cycle life). The root cause of this distinction is that the initial stripping or plating gives rise to the different morphological evolution from pristine Zn: the initial stripping process for S-Zn leads to the formation of cracks, thus triggering dendritic Zn growth from the cracks, while the initial plating for P-Zn generates a relatively uniform plating behaviour due to the generation of a uniform lamellar structure (Fig. 4j).

### Electrochemical and physicochemical characterizations of Zn metal electrodeposition on various substrates

With the phenomenon observed in the previous experiments, P-Zn demonstrates a more uniform Zn morphology evolution. However, the RZBs are initially (after assembly) in a charged state. The first step is discharge, where the Zn anode donates Zn^2+^ and the cathode receives Zn^2+^, corresponding to a stripping process at the Zn anode. Based on the analyses in the last two sections, predeposition should be effective for alleviating the dendrite cluster formation.

**Fig. 5:**
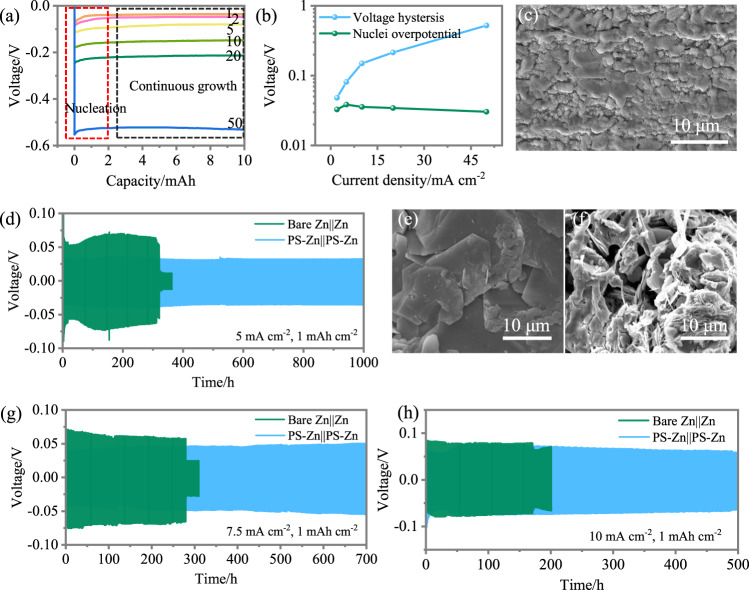
Electrochemical and microscopy characterizations of Zn metal electrodes. **a** Voltage profile of Zn deposition under various current densities (mA cm^−2^); **b** voltage hysteresis and nuclei overpotential vs. current density; **c** ex situ SEM micrograph of the P-Zn electrode cycled at 50 mA cm^−2^; **d** voltage–time profiles of bare Zn||Zn and PD-Zn||PD-Zn symmetric cells at current densities of 5 mA cm^−2^, 7.5 mA cm^−2^, and 10 mA cm^−2^; **e** morphology of PD-Zn electrode after 1000 h cycling at 5 mA cm^−2^; **f** morphology of bare Zn within the short-circuited cell after 360 h cycling at 5 mA cm^−2^; voltage–time profiles of bare Zn||Zn and PD-Zn||PD-Zn symmetric cells at current densities of **g** 7.5 mA cm^−2^ and **h** 10 mA cm^−2^.

The deposition behaviour is first investigated. The deposition of Zn is composed of two parts, as shown in Fig. 5a: nucleation and continuous growth. Both processes are strongly influenced by the current density. The deposition morphology on the Cu substrate (Supplementary Fig. 9) was examined by SEM observation. As demonstrated in Supplementary Fig. 10, the nuclei size declines with increasing current density, and the nuclei density is proportional to the current density. The deposited Zn is in the form of aggregates (Supplementary Fig. 10a–h) at a small current density. Comparatively, the deposited Zn at 20 and 50 mA cm^−2^ show higher uniformity and covers the whole substrate (Supplementary Fig. 10i–l). In addition to the morphology, the crystallographic orientation of the deposited Zn is also associated with the current density. The deposited Zn shows colour changes from black with low current density to grey with higher current density with more homogenous distribution (Supplementary Fig. 11a), reflecting the Zn deposited at a higher current density (above 10 mA cm^−2^)^32^. The intensities of (002), and (103) reflections (Supplementary Fig. 11b) increase as the current density increases with 0–30^o^ alignment to the substrate, which is less likely to form dendrites^33^, and the initial crystallographic orientation exerts an impact on Zn deposition in the consequent cycles^34^. The voltage hysteresis (i.e., the voltage level in the continuous growth” stage) is proportional to the current density increase, while the nucleation overpotential (i.e., the extra potential between the nucleation stage and continuous growth stage) shows the limited change (Fig. 5b). This indicates that the nuclei density is associated with voltage hysteresis^35,36^. Similar phenomena were found when switching the substrates to stainless steel (SS) and Ti in terms of the correlation between homogeneity and current density (Supplementary Figs. 12–15), and the Zn power electrode also followed a similar trend (Supplementary Fig. 16). Combining all the results of the morphological uniformity and crystallographic orientation, a current density higher than 20 mA cm^−2^ should be considered to fabricate the predeposition layer.

To create PD-Zn, an areal capacity of 0.3 mAh cm^−2^ as a predeposited layer was taken as an example. Compared with the Zn deposited at 20 mA cm^−2^ (Supplementary Fig. 17), the plated Zn deposited at 50 mA cm^−2^ shows a more uniform morphology with a compact layer structure (Fig. 5c). Current densities higher than 100 mA cm^−2^ can induce irreversible Zn-ion depletion in the electrolyte solution and further cause dendritic and heterogeneous morphology (Supplementary Fig. 18). Additionally, among the predeposition currents (10, 20, 50 mA cm^−2^), the nitrogen adsorption–desorption and electrochemical impedance spectroscopy (EIS) measurements of the electrode materials suggest that the predeposited layer fabricated at 50 mA cm^−2^ is more compact (Supplementary Fig. 19a, Supplementary Table 1, and Supplementary Note 1) and can facilitate the charge transfer process (Supplementary Fig. 19b, Supplementary Table 2, and Supplementary Note 2). An ideal Zn anode should first have an initial uniform morphology that avoids the protrusion effect (i.e., when the protrusion of the Zn metal dendritic depositions incorporate additional Zn ions due to the enhanced electric field), resulting in a beneficial crystallographic texture to enable chemical and structural stability. Finally, a sufficient Zn ion supply is necessary to avoid ion depletion and dendrite formation. Here, in this work, we are able to satisfy the first two conditions, and the last factor can be avoided when the cells are cycled at a reasonable current density and deposition amount (e.g., 5 mA cm^−2^, 1 mAh cm^−2^). This PD-Zn fabricated at 50 mA cm^−2^ serves as a viable choice for subsequent uniform plating/stripping (Supplementary Fig. 20)^6^.

To further test the long-term cyclability, symmetric Zn||Zn cells were assembled and tested. The untreated Zn metal electrodes cycled at a current density of 2 mA cm^−2^ led to cell failure after 50 h with a voltage hysteresis of approximately 70 mV (Supplementary Fig. 21). The PD-Zn||PD-Zn cell shows more than 1000 h of cycling with a stable voltage profile with a voltage hysteresis of 52 mV, implying that PD-Zn is kinetically faster than bare Zn with a uniform stripping/plating. This can also be validated by the EIS measurements, as disclosed in Supplementary Fig. 22 and Supplementary Table 3. The *R*_ct_ for PD-Zn||PD-Zn is approximately 20 Ω, which is half the value for the bare Zn||Zn cell (127 Ω). This may result from the regular texture of PD-Zn, which is beneficial for charge transfer^37^. Additionally, for the PD-Zn||PD-Zn cell, the voltage profile after 1000 h is similar to the initial cycles (Supplementary Fig. 23). When the current density increases to 5 mA cm^−2^, the bare Zn||Zn cell shows a short lifespan of approximately 300 h before the voltage drop and short circuit (Fig. 5d), while PD-Zn||PD-Zn has a long lifespan of over 1000 h. The voltage hysteresis is also decreased from 141 to 70 mV by applying this predeposition strategy, suggesting stable stripping and plating. The PD-Zn electrode after 1000 h cycling that of at 5 mA cm^−2^ shows a uniform morphology with blocks of hexagonal lamellae (Fig. 5e). In contrast, the untreated bare Zn after short circuits shows a dendritic morphology (Fig. 5f), leading to the short circuit. Further cycling at higher current density of 7.5 mA cm^−2^ (Fig. 5g) and 10 mA cm^−2^ (Fig. 5h and Supplementary Fig. 24) was carried out, and the PD-Zn||PD-Zn cells all demonstrated a longer lifespan and marginally lower voltage hysteresis, confirming the stability of the PD-Zn electrode. This pre-deposition strategy can improve the stability of Zn anode without the synthesis of complex materials or incorporating intricate preparation methods, and the active and uniform predeposition layer can induce the consequent uniform deposition (Supplementary Fig. 9). Notably, the lifespan of the bare Zn||Zn cell did not decrease prominently as the current density increased (Fig. 5). This can be ascribed to the longer Sand’s time (Supplementary Fig. 25) and the dense and compact Zn layer formed under high current densities (Supplementary Fig. 26)^36^. This leads to the lifespan difference between PD-Zn||PD-Zn and bare Zn||Zn cells being more prominent when cycled at a relatively low current density.

The effectiveness of this predeposition strategy is also validated by adopting a thinner nonwoven separator with a lifespan of over 1000 h for the symmetric cell (Supplementary Fig. 27 and Supplementary Note 3). Furthermore, the correlation between current density and homogeneity is verified in other electrolytes, such as 1 M zinc bis(trifluoromethylsulfonyl)imide (Zn(TFSI)_2_)/H_2_O and 1 M Zn(TFSI)_2_/H_2_O:dimethoxyethane (DME) (1:1 by volume) (Supplementary Figs. 28, 29, 30, 31 and Supplementary Note 4). Therefore, this strategy can also be compatible with other electrolyte systems. Fundamentally, the predeposition strategy can be categorized into artificial interface modification and could affect the consequent morphology evolution. This strategy is simple and effective, and the PD-Zn||PD-Zn symmetric cell is well-positioned compared with the recent Zn metal anode modification (Supplementary Table 4 and Supplementary Note 5). Additionally, this strategy can serve as a supplementary approach along with electrolyte modification to further enhance the anode performance: taking the additive added (0.05 M KPF_6_) as an example, PD-Zn||PD-Zn shows a prolonged lifespan of over 1000 h (Supplementary Fig. 32).

### Electrochemical characterizations of predeposited Zn metal electrodes in combination with MnO_2_-based electrodes

**Fig. 6:**
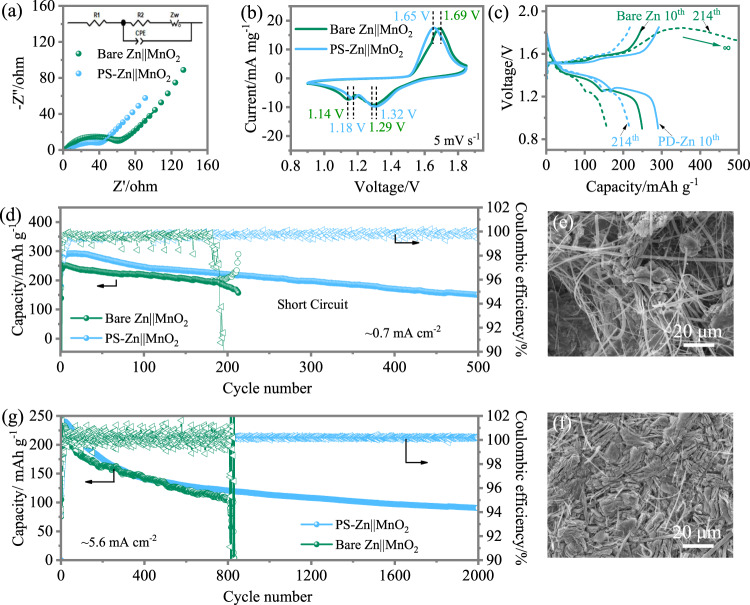
Electrochemical characterizations of aqueous Zn||MnO_2_ coin cells. **a**, **b** EIS and CV curves of the fresh PD-Zn||MnO_2_ and bare Zn||MnO_2_ cells; **c**, **d** GCD profiles and cycling performance at a specific current of 0.5 A g^−1^ with a positive electrode loading mass of 1.4 mg cm^− 2^; **e**, **f** ex situ SEM micrograph of the disassembled bare Zn after 214 cycles and PD-Zn after 500 cycles; **g**. cycling performance of PD-Zn||MnO_2_ under a current density of 1 A g^−1^ with a positive electrode loading mass of 5.6 mg cm^−2^.

Full cells of bare Zn and PD-Zn coupled with δ-MnO_2_ (Supplementary Fig. 33) are assembled to evaluate the influences of the S-Zn and P-Zn anodes on the full cell performance. The Nyquist plot of the initial cells of bare Zn||MnO_2_ and PD-Zn||MnO_2_ enables the extrapolation of the charge transfer resistance (*R*_ct_). The *R*_ct_ of the PD-Zn||MnO_2_ cell with 40 Ω is much less than that of the bare Zn||MnO_2_ cell with 65 Ω, as shown in the Nyquist plot in Fig. 6a and the fit data reported in Supplementary Table 5, suggesting that PD-Zn is kinetically favourable for charge transfer. The cyclic voltammetry (CV) curves in Fig. 6b support the EIS speculations. The oxidative peak (1.65 V) of PD-Zn||MnO_2_ is lower than that of bare Zn||MnO_2_ (1.69 V), and the reductive peaks of PD-Zn||MnO_2_ are all higher (1.32 V vs. 1.29 V, 1.18 V vs. 1.14 V) than that of bare Zn||MnO_2_. These results suggest less polarization of the PD-Zn anode compared with the bare Zn anode. A slightly higher capacity for PD-Zn||MnO_2_ was demonstrated through further cycling performance (Fig. 6c, d) with the specific current of 0.5 A g^−1^. After 200 cycles, the charge and discharge capacity of bare Zn||MnO_2_ deviate gradually, and the coulombic efficiency drops accordingly. The cell fails during the 214th cycle, as shown in Fig. 6c. The charge curve of the green dashed line shows infinite charge capacity, and the voltage cannot reach the cut-off voltage (1.85 V), which is normally viewed as a short circuit. In contrast, the PD-Zn||MnO_2_ cell shows a similar cycling trend without the occurrence of a short circuit (Fig. 6d). The plateaus of the galvanostatic charge–discharge (GCD) curves of PD-Zn||MnO_2_ are also consistent with the CV peaks (Fig. 6c). After 500 cycles, the cells were disassembled for further analysis. As shown in Fig. 6e, the morphology of bare Zn is unregulated with sharp tips, which is the culprit for the short circuit. In contrast, PD-Zn exhibits a flat surface with uniform and compact Zn deposition (Fig. 6f). High mass loading (5.6 mg cm^−2^) of the cathode (MnO_2_) was used to further test the stability of the PD-Zn electrodes. No short circuit was observed within 2000 cycles (Fig. 6g). The cycling performances obtained for the PD-Zn||MnO_2_ cells are well positioned compared with recent literature (see Supplementary Table 6).

To demonstrate the applicability of the predeposited Zn anode strategy, activated carbon-based (C) positive electrodes have been taken into account. Further Zn||C cells (Supplementary Fig. 34) were introduced to evaluate the anode performance, and the results are illustrated in Supplementary Fig. 35. The PD-Zn||C cell demonstrates a lifespan exceeding 10,000 cycles without a short circuit. In contrast, the bare Zn||C cell shows a similar capacity deviation, coulombic efficiency decline, and failure after 3642 cycles (Supplementary Fig. 36).

In summary, the dendrite issue of the Zn anode is detrimental to cell performance. Previous studies have focused on the plating behaviour without consideration of the initial stripping process. This work investigated the voltage profile and morphology evolution of P-Zn and S-Zn within a symmetric Zn||Zn cell separately. We demonstrate that the stability performance of the Zn anode was affected by not only the operating conditions, but also the initial stripping/plating. The Zn electrode that was initially plated in the first cycle demonstrated a more uniform morphology in subsequent cycles. In contrast, severe dendrites formed on the pit position for an initially stripped Zn electrode. Based on these analyses, a predeposition strategy is proposed to induce uniform Zn plating/stripping. The corresponding Zn||MnO_2_ cell delivers a better performance without the sign of a short circuit, and the Zn||C cell exhibits 10,000 cycles of stable cycling. This work has provided insight into both the stripping and plating processes of a Zn anode, demonstrating their equal importance in terms of dendrite formation. This work can serve as a starting point to inspire researchers to investigate the stripping process of metal anodes.

## Methods

### Materials

ZnSO_4_.7H_2_O (analytical reagent), dimethoxyethane (DME, analytical reagent), N-methylpyrrolidone (NMP, analytical reagent), isopropyl alcohol (analytical reagent), Mn(Ac)_2_ (99.99%), and KPF_6_ (99,98%) were purchased from Aladdin. Zinc bis(trifluoromethylsulfonyl)imide (Zn(TFSI)_2_, 95%) and hydrazine hydrate solution (N_2_H_4_.H_2_O, 80%, Sigma-Aldrich, for synthesis) were purchased from Sigma-Aldrich. Cu foil (99.8%, 9 μm), Ti foil (99.9%, 20 μm), SS foil (99.99%, 20 μm), polyvinylidene difluoride (PVDF-900, 99.5%), Ketjen black ECP-600JD (conductive agent), Carbon cloth (CC, WIS1010, thickness: 0.38 mm) and PTFE emulsion (60 wt%) were purchased from CANRD. Ti mesh (100 mesh, 99.9%) was obtained from Kangwei Metal. Zn foil (200 μm and 80 μm, 99.99%) was purchased from Chenshuo Metal. The active carbon powder (YP-50F) with a particle size of 5–20 μm and a surface area of 1500–1800 m^2^ g^−1^ was obtained from KURARAY.

### Fabrication of PD-Zn

Initially, a symmetric cell (pouch cell) with two Zn foils (size: 1 cm × 1 cm, 0.1 mm, 99.99%, CHENSHUO Metal) was assembled with glass fibre (Whatman, GF/D, 260 μm, average pore size: 2.7 μm) as the separator and 2 M ZnSO_4_ (100 μL) as the electrolyte. One side was deposited with 0.3 mAh cm^−2^ Zn at a current density of 50 mA cm^−2^, denoted PD-Zn. The as-fabricated PD-Zn was washed with water and ethanol three times and dried in air before being used in symmetric cells and Zn||MnO_2_ cells.

### Zn powder electrode

Zn powders (average particle size: 5 μm, Mingchuang New Material Co. Ltd) were mixed with PVDF as a binder at a mass ratio of 97:3 (total mass of 1 g). The powder mixture was ground in a mortar for 30 min, further NMP was added to form a homogeneous slurry, and the slurry was stirred overnight before it was coated on Cu foil by a surgical blade (120 μm). The average loading of Zn powder was 10 mg cm^−2^.

### Synthesis of δ-MnO_2_

Hydrazine hydrate solution (10 mL, N_2_H_4_.H_2_O, 80%, Sigma-Aldrich, for synthesis) was added to 200 mL Mn(Ac)_2_ solution (4 mmol L^−1^) dropwise under constant stirring. The mixture was reacted for 5 min before being transferred into a Teflon-lined stainless-steel autoclave, and the temperature was maintained at 180 °C for 12 h. The white precipitate Mn(OH)_2_ was collected after water filtration three times. Mn(OH)2 (1.0 g) was weighed and dispersed with 250 mL water to form a uniform dispersion by sonicating for 10 min. Then, 50 mL of NaClO solution was added to the Mn(OH)_2_ dispersion dropwise under constant stirring. After that, the mixture was stirred for another 24 h, and the precipitate (δ-MnO_2_ nanoplates) was collected after water filtration three times and dried in a vacuum oven overnight.

### Characterizations

Scanning electron microscopy (SEM, FEI Quanta 450 FEG SEM) was applied to observe the Zn and MnO_2_ morphology. A Bruker D2 Phaser with Cu Kα radiation (λ = 0.15418 nm) operating at 30 kV and 10 Ma was adopted to obtain XRD curves. Optical microscopy (OM, Olympus, SC180) was performed with in situ homemade cells to observe the zinc morphology. The specific surface area was measured with N_2_ adsorption–desorption measurements (Micromeritics, ASAP 2020) using the Brunauer–Emmett–Teller (BET) method.

### Cell assembly

Symmetric cells (two-electrode cell, coin cell 2032) were assembled by using PD-Zn or bare Zn electrodes (thickness of 80 μm, size: 1 × 1 cm) with 2 M ZnSO_4_ as the electrolyte and Whatman glass fibre (GF/D, 260 μm, average pore size: 2.7 μm) as the separator. Thin separator experiments were also conducted by using nonwoven paper (thickness: 113 μm, average diameter: 20 μm, 55% cellulose and 45% polyester, Jiangsu Wuchen Technology). For the low loading mass MnO_2_ electrode (approximately 1.4 mg cm^−2^) and active carbon electrode (approximately 1.5 mg cm^−2^), the cathode slurry was composed of δ-MnO_2_ or active carbon, PVDF, or carbon black at a ratio of 7:1:2. First, the powders of δ-MnO_2_ or active carbon, PVDF, and carbon black were mixed in a mortar for 30 min, and NMP was added to form a homogeneous slurry and stirred overnight. The slurry was then coated on carbon cloth (WIS1010, thickness: 0.38 mm) and dried at 60 °C in a vacuum oven overnight before use. For the high loading mass MnO_2_ electrode (approximately 5.6 mg cm^−2^), the cathode slurry was composed of δ-MnO_2_, PTFE, and carbon black at a ratio of 7:1:2. The mixture was ground in a mortar for 5 min, and isopropyl alcohol was added to further grind the mixture into a dough. The dough was rolled into the Ti mesh substrate and dried at 60 °C in a vacuum oven overnight before use. The cells used PD-Zn or bare Zn as the anode and MnO_2_ or carbon as the cathode. The electrodes were employed without a further calendering process. Full Zn||MnO_2_ cells (two-electrode cell, coin cell 2032) were assembled by using PD-Zn or bare Zn electrodes (thickness of 80 μm, size: 1 × 1 cm) and MnO_2_ electrodes (1 × 1 cm) with 2 M ZnSO_4_ and 0.1 MnSO_4_ aqueous solution as the electrolyte and Whatman glass fibre (GF/D, 260 μm, average pore size: 2.7 μm) as the separator. Full Zn||C cells (two-electrode cell, coin cell 2032) were assembled by using PD-Zn or bare Zn electrodes (thickness of 80 μm, size: 1 × 1 cm) and an active carbon electrode (1 × 1 cm) with 2 M ZnSO_4_ aqueous solution as the electrolyte and Whatman glass fibre (GF/D, 260 μm, average pore size: 2.7 μm) as the separator. The electrolyte amount was controlled to 100 μL per cell for all cells. 1–3 cells were cycled for each electrochemical experiment. For Zn||MnO_2_ and Zn||C cells, the specific current and specific capacity values refer to the mass of active material in the positive electrode.

### Preparation of ex situ samples

The ex situ Zn samples with different states in Fig. 4 were obtained by using two Zn electrodes (200 μm for better fixation, 99.99%) in a glass cell (two electrodes). After several plating/stripping processes, the Zn electrodes with different states were removed from the cell, washed with deionized water three times to remove the residual salts, washed with ethanol three times, and dried at ambient temperature overnight before further characterization. The samples of N_2_ adsorption–desorption measurements were obtained by using Cu foil (1 × 1 cm) and Zn foil (80 μm, 1 × 1 cm) as two electrodes in a glass cell with 2 M ZnSO_4_ aqueous solution as the electrolyte, and 10 mAh Zn was deposited on Cu foil. The Cu foil with deposited Zn was washed 3 times with water, washed 3 times with ethanol and dried in a vacuum oven at 60 °C overnight. The Cu foil was cut into slices for N_2_ adsorption–desorption measurements.

### Electrochemical measurements

EIS was recorded by an electrochemical workstation (CHI 760E) under quasi-stationary potential mode within the frequency range of 100 kHz–0.01 Hz for the full cell (data points: 85) and 100 kHz–0.1 Hz (data points: 73) for the symmetric cell by applying a 5 mV AC oscillation. CV curves were acquired with the same CHI 760E workstation. The cycling performance and rate performance of both asymmetric and full cells were validated with a LAND or Neware battery test system. The voltage windows for the Zn||MnO_2_ and Zn||C cells were 0.85–1.85 V and 0.3–1.85 V, respectively. The specific capacity value of full cells is calculated based on the mass of MnO_2_ or active carbon. Voltage profiles for the separate electrodes in the symmetric cell were recorded with two LAND channels at the same time. All electrochemical measurements were performed in the open air of a thermostatic room (25 ± 1 °C).

### Reporting summary

Further information on research design is available in the Nature Research Reporting Summary linked to this article.

## Supplementary information

Supplementary Information

Reporting Summary

## Data Availability

The data that support the findings of this study are available within the text including the Methods, and Supplementary information. Raw datasets related to the current work are available from the corresponding author on reasonable request.
