# Supplementary material for: Tailoring the metal electrode morphology via electrochemical protocol optimization for long-lasting aqueous zinc batteries
Source: Nat Commun. 2022 Jun 27;13:3699. doi: 10.1038/s41467-022-31461-7 (PMC9237080; doi:10.1038/s41467-022-31461-7)
Supplement: Supplementary file 1 — Supplementary Information [file 41467_2022_31461_MOESM1_ESM.pdf]

Supplementary Information

Tailoring the metal electrode morphology via electrochemical protocol optimization for long-lasting aqueous zinc batteries

Qing Li[1], *Ao Chen*[1], *Donghong Wang*[2], *Yuwei Zhao*[1], *Xiaoqi Wang*[3], *Xu Jin*[3], *Bo Xiong*[3], *Chunyi Zhi**[1,2]

1. Department of Materials Science and Engineering, City University of Hong Kong, 83 Tat Chee Avenue, Kowloon, Hong Kong 999077, China.
   E-mail: cy.zhi@cityu.edu.hk

2. Hong Kong Center for Cerebro-Cardiovascular Health Engineering (COCHE), Shatin, NT, HKSAR, China

3. Research Institute of Petroleum Exploration & Development (RIPED), PetroChina, Research Center of New Energy, No. 20 Xueyuan Road Haidian District, Beijing, 100083, P. R. China

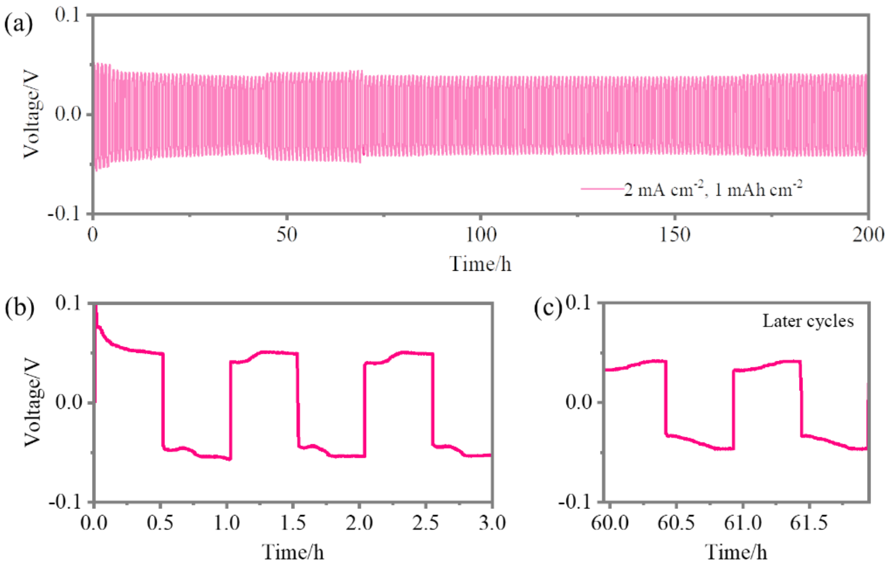

Supplementary Figure 1. The voltage-time profile of a symmetric cell combining two Zn foil electrodes in the glass cell at ambient temperature (25°C).

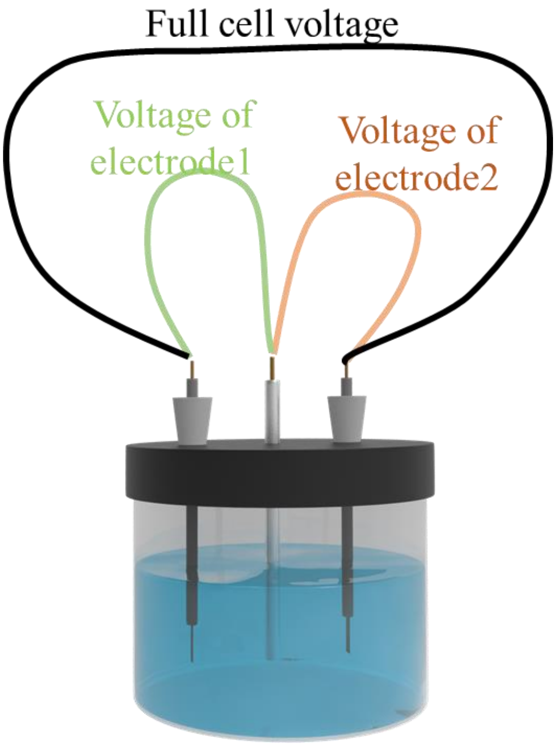

Supplementary Figure 2. The schematic of the voltage monitoring for two electrodes in a symmetric cell (glass cell). Electrodes 1 and 2 (two Zn foils) with 1x1 cm size were placed face to face with a distance of 1 cm, the reference electrode was a Zn foil 0.5 x

1cm, and the electrolyte was 2M $ZnSO_4$ solution. The test was conducted at ambient temperature (25 °C).

The voltage of electrodes 1 and 2 vs reference electrode was recorded with two LAND channels, and the voltage between electrodes 1 and 2 was also recorded via another land channel. With this configuration, the voltage of a separate electrode can be monitored promptly during the plating and stripping process.

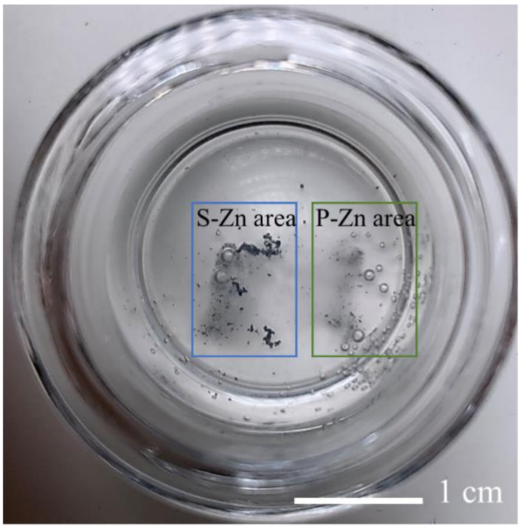

Supplementary Figure 3. The photographic picture of dead Zn at the bottom of a cell.

The left area within the blue square (area at the bottom of the electrode with initial stripping) shows more dead Zn (falling from the electrode) than the right area within the green square (area at the bottom of the electrode with initial stripping).

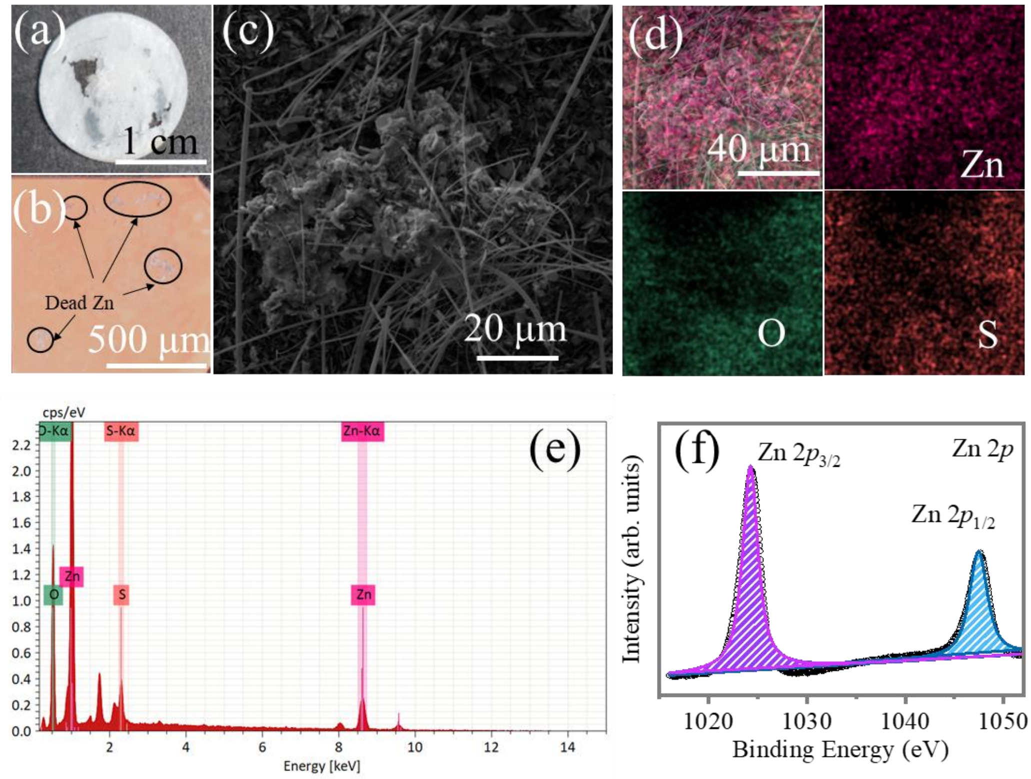

Supplementary Figure 4. Physicochemical characterization of electrochemically deposited Zn on a Cu substrate. The photographic picture of the a. cycled separator and b. Cu substrate after Zn being fully stripped from Cu substrate; c. the SEM images, d. EDS mapping, e. EDS spectra, f. XPS spectra of the dead Zn.

To demonstrate the existence of dead Zn, we conducted the experiment using the Zn||Cu asymmetric cell. A certain amount of Zn (1 mAh cm^{-2}) was first deposited into the Cu substrate and followed by the stripping process with a cut-off voltage of 0.8 V. The cell was disassembled after 10 cycles and the Cu electrode is in a fully stripped state. The photographic picture show the color of the remaining Zn elements existing in both the separator and Cu substrate (Supplementary Figure 4 a,b), and the remaining Zn cannot be stripped when reaching the cut-off voltage. This can be regarded as inactive Zn. The SEM, EDS, and XPS characterization all further proves the existence of elementary Zn remaining in the Cu substrate, which can be ascribed to the “dead Zn” under the current definition (Supplementary Figure 4 c,d,e,f).

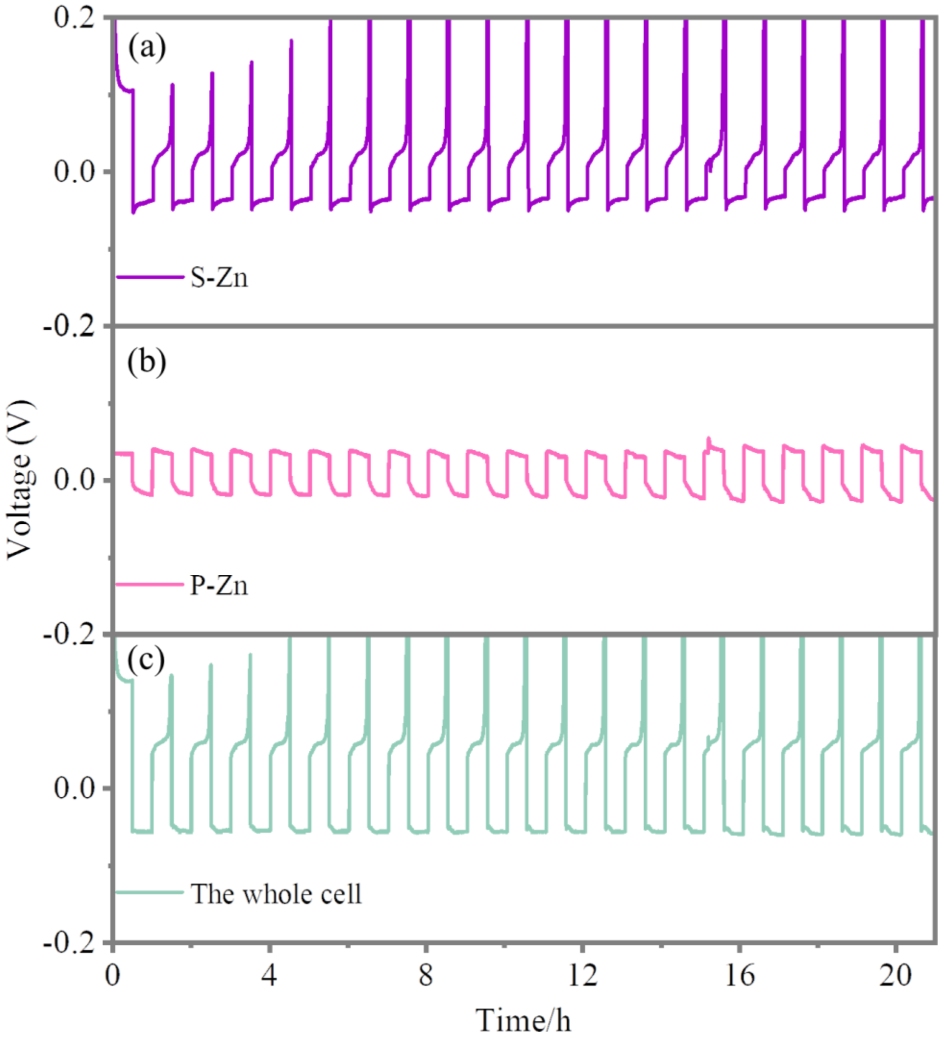

Supplementary Figure 5. The time-voltage profile for a. S-Zn power electrode and b. P-Zn power electrode, and c. the whole systematic cell (glass cell) cycled at 2 mA cm^{-2}, 1 mAh cm^{-2}, and ambient temperature (25°C).

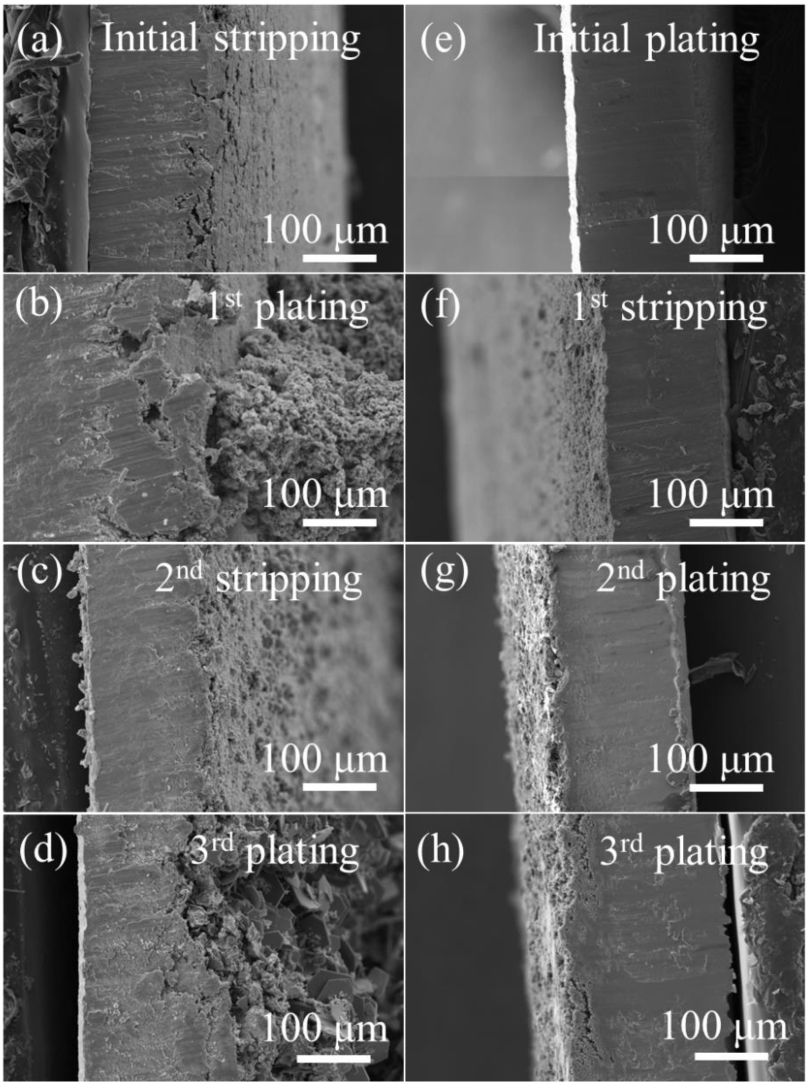

Supplementary Figure 6. The SEM images of a. the initial S-Zn, cycled S-Zn after b. the 1st plating, c. the 2nd stripping, d. and the 3rd plating at an angle of 90°; the SEM images of e. the P-Zn, cycled P-Zn after f. the 1st stripping, g. the 2nd plating, and h. the 3rd stripping at an angle of 90°. The cycling of the Zn electrodes was conducted at 10 mA cm^{-2}, 5 mAh cm^{-2}, and room temperature in glass cells.

As shown in Supplementary Figure 6a, cracks have been detected in the initial stripping of the S-Zn. At the consequent plating, the Zn dendrite cluster appears (Supplementary Figure 6b). Some mossy Zn has been witnessed at the 2nd stripping (Supplementary Figure 6c). After the 3rd plating, the Zn morphology is more heterogeneous, with big flakes and mossy Zn coexisting. While for the P-Zn, the initial plating is more uniform (Supplementary Figure 6e), and less mossy Zn is observed after the following stripping process (Supplementary Figure 6f). The morphology of the

following 2nd and 3rd Zn plating is also homogenous (Supplementary Figure 6g,h), and this is in line with the side view SEM observation at an angle of 45° (Figure 3).

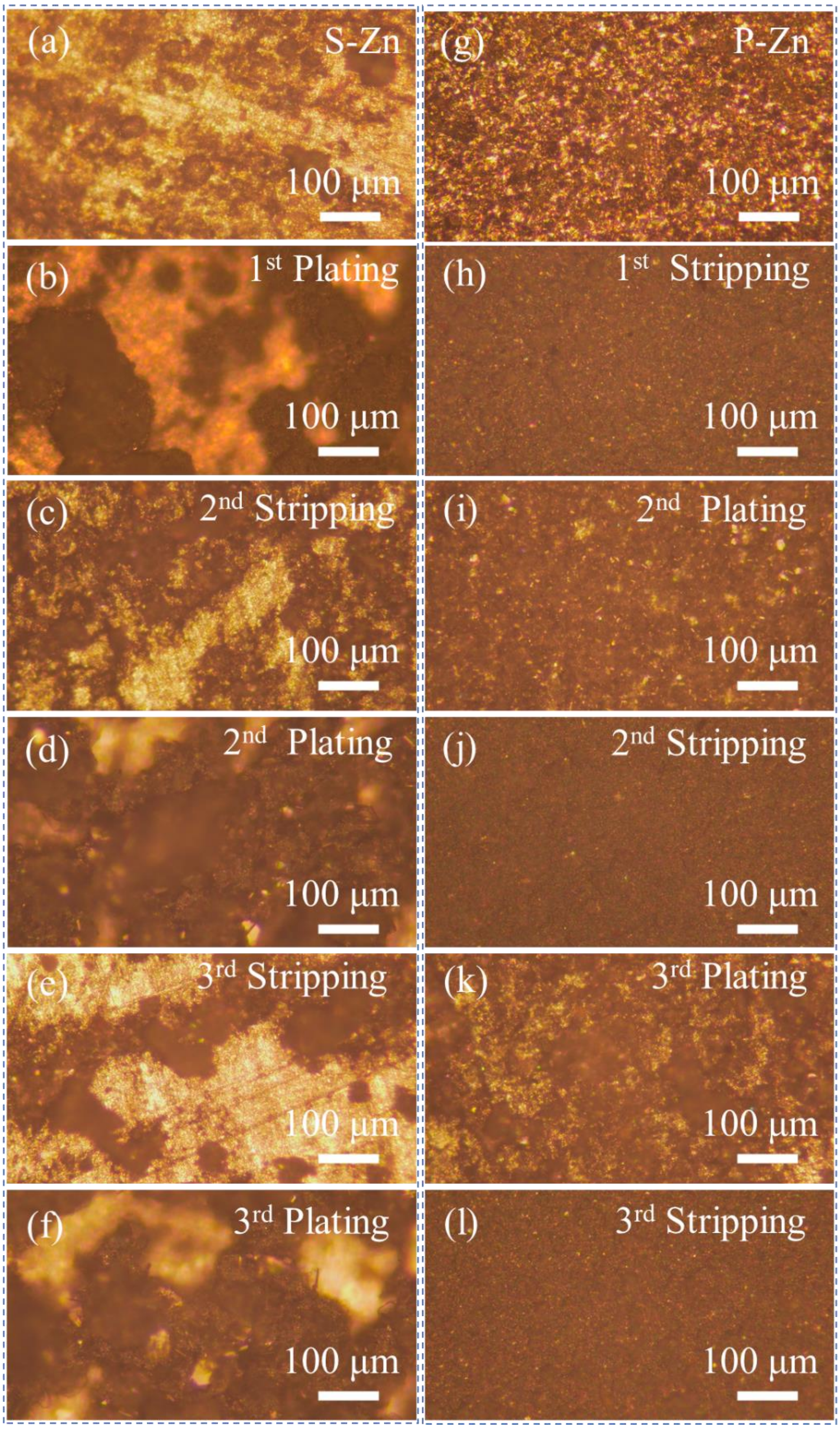

Supplementary Figure 7. The microscopy figure (top view) of a. initial S-Zn , b.cycled S-Zn after the 1st plating, c. the 2nd stripping, d. 3rd plating, e. 3rd stripping, e. 3rd plating;

and f. P-Zn ; The microscopy figure of g. initial P-Zn, h.cycled P-Zn after the 1st plating, h. the 2nd stripping, i. 3rd plating, j. 3rd stripping, k. 3rd plating, l. and P-Zn. The cycling of the Zn electrodes was conducted at 10 mA cm^{-2}, 5 mAh cm^{-2}, and room temperature in glass cells.

As shown in Supplementary Figure 7a-f, the heterogeneity of S-Zn is enlarged with the stripping/plating process. While for the P-Zn in Supplementary Figure 7g-l, the Zn surface demonstrates a much higher level of homogeneity.

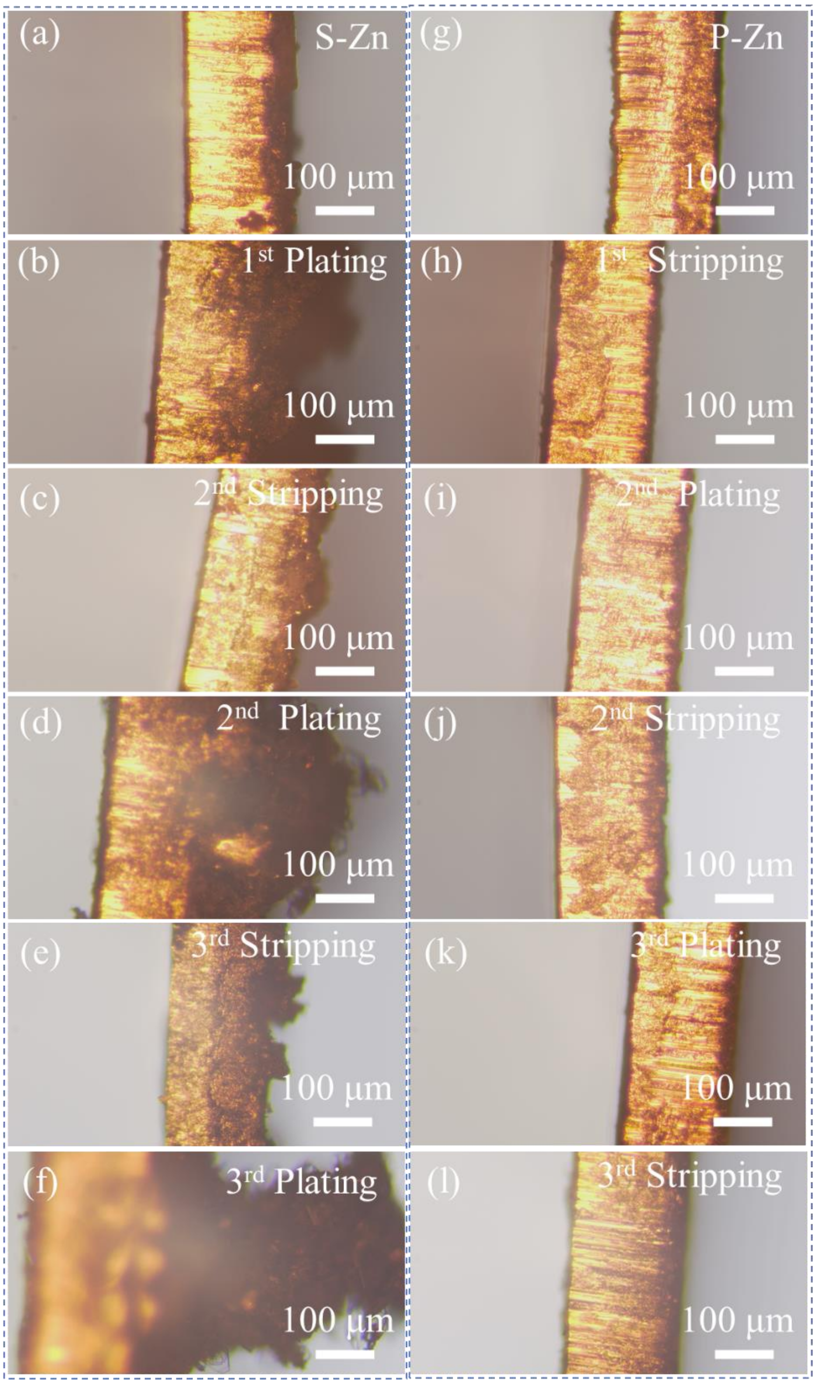

Supplementary Figure 8. The microscopy figure (cross-section view) of a. initial S-Zn, cycled S-Zn after b. the 1st plating, c. the 2nd stripping, d. 3rd plating, e. 3rd stripping, f. 3rd plating; The microscopy figure of g. initial P-Zn, cycled P-Zn after h. the 1st plating , i. the 2nd stripping, j. 3rd plating, k. 3rd stripping, l. 3rd plating. The cycling of the Zn electrodes was conducted at 10 mA cm^{-2}, 5 mAh cm^{-2}, and room temperature in glass cells.

As shown in Supplementary Figure 8a-f, pits are observed in S-Zn, and then the

Zn deposit on the positions of pits forming the Zn dendrite cluster. These dendrites are not fully removed in the subsequent stripping, and the Zn dendrites grow higher (more than 300 μm) in the following plating process. Repeating this cycle, the Zn deposits turn into huger Zn dendrites. On the contrary, the P-Zn (Supplementary Figure 8g-l) is more uniform in the initial plating process, and the following stripping/plating process is also homogenous (only slight heterogeneity can be found).

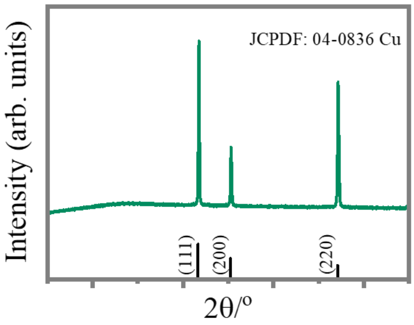

Supplementary Figure 9. The X-ray diffractogram of commercial Cu foil used in the electrochemical experiments.

The X-ray diffractograms shows that the Cu foil is polycrystal Cu, and the main contributing planes are (111), (200), (220).

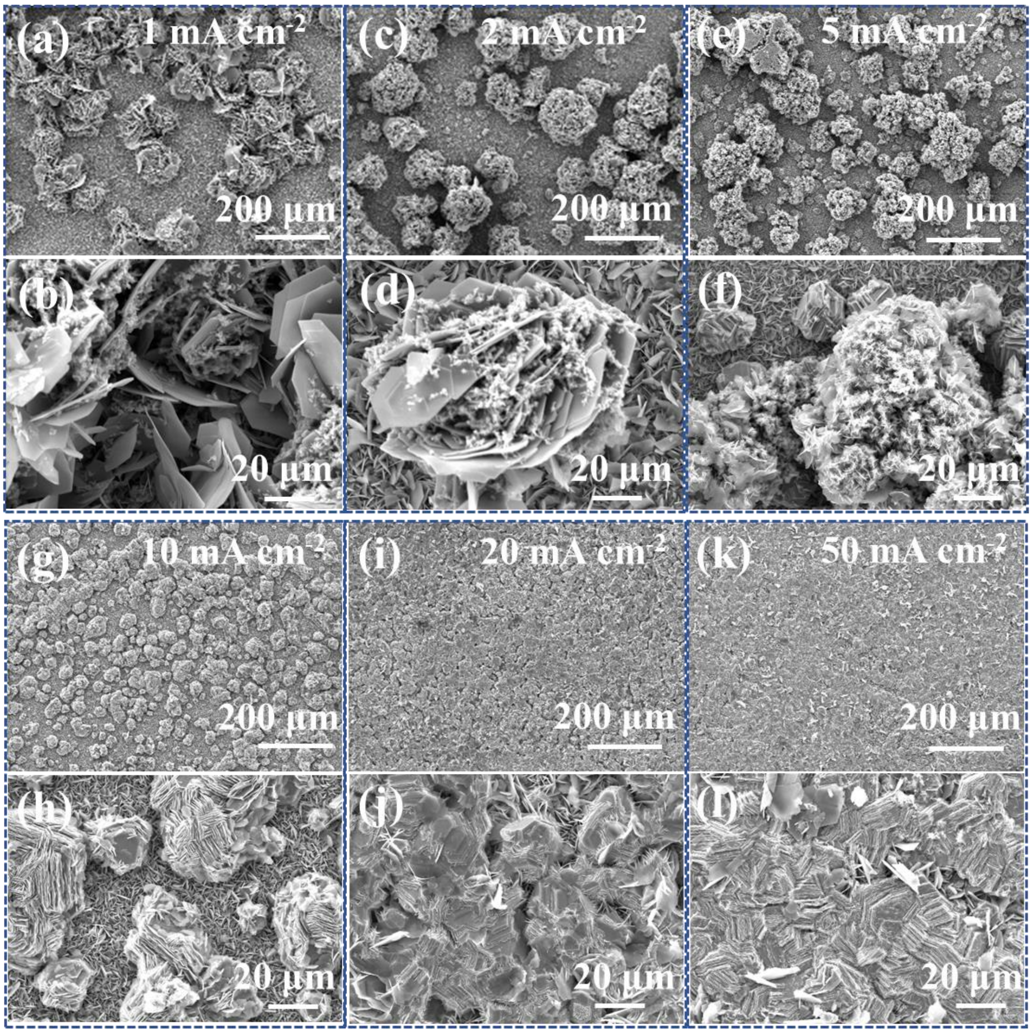

Supplementary Figure 10. The SEM images of Zn morphology deposited on a Cu substrate at different current densities: a,b. 1, c,d. 2, e,f. 5, g,h. 10, i,j. 20, k,l. 50 mA cm^{-2}. The deposition process was conducted in glass cell at ambient temperature (25 ℃) with 2M $ZnSO_4$ aqueous electrolyte.

The current density has a significant influence on the deposited Zn morphology. As shown in Supplementary Figure 10, the nucleation size is decreased with the increase of current density, and the nucleation density is proportional to the current density. Comparatively, the deposited Zn in 50 mA cm^{-2} shows the highest uniformity.

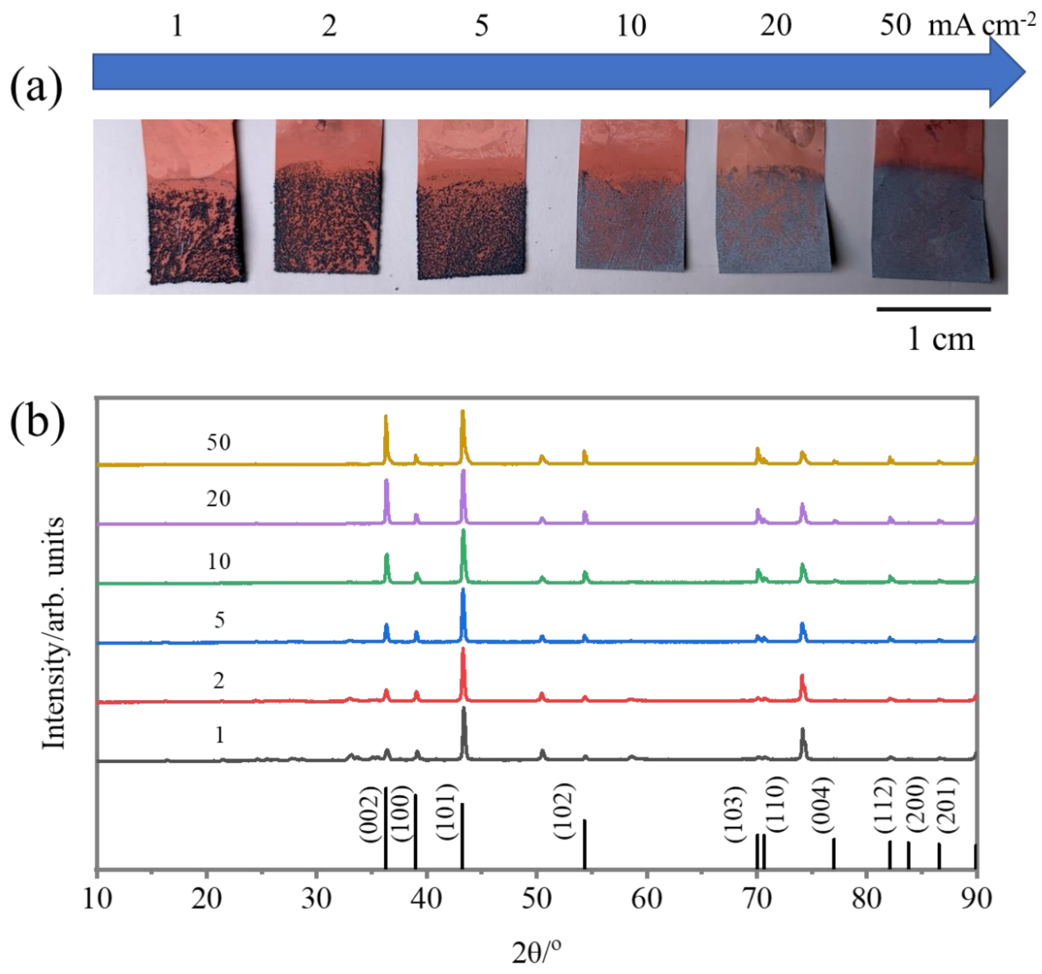

Supplementary Figure 11. a. The photographic picture and b. normalized X-ray diffractograms of the deposited Zn on Cu substrate at different current densities. The deposition process was conducted in glass cell at ambient temperature (25 °C) with 2M $ZnSO_4$ aqueous solution as electrolyte.

The photographic picture of deposited Zn under various current densities shows color changes from black to grey with the current density increase. This can indicate that the Zn deposited at small current densities is mossy and irregulated, while the Zn deposited at the higher current density above 10 mA cm^{-2} is closely packed, and more compact. Beyond that, the uniformity of the deposit is also diverse, and the Zn plated at the higher current density shows a uniform distribution. Further XRD is applied to verify the hypothesis and get more information on the crystallographic orientation. As shown in Supplementary Figure 11b, the relative intensities of (002) and (103) are

enhanced with the increase of the current density. These two peaks are considered as the basal types and exhibited 0-30° alignment to the substrate, which is less likely to form dendrites and destroy batteries[1]. Since the initial crystallographic orientation will affect the Zn deposition in the consequent cycle [2], the relatively high intensity of Zn (002) may be attributed to the substrate impact and the lattice match between Cu(111) and Zn(002).

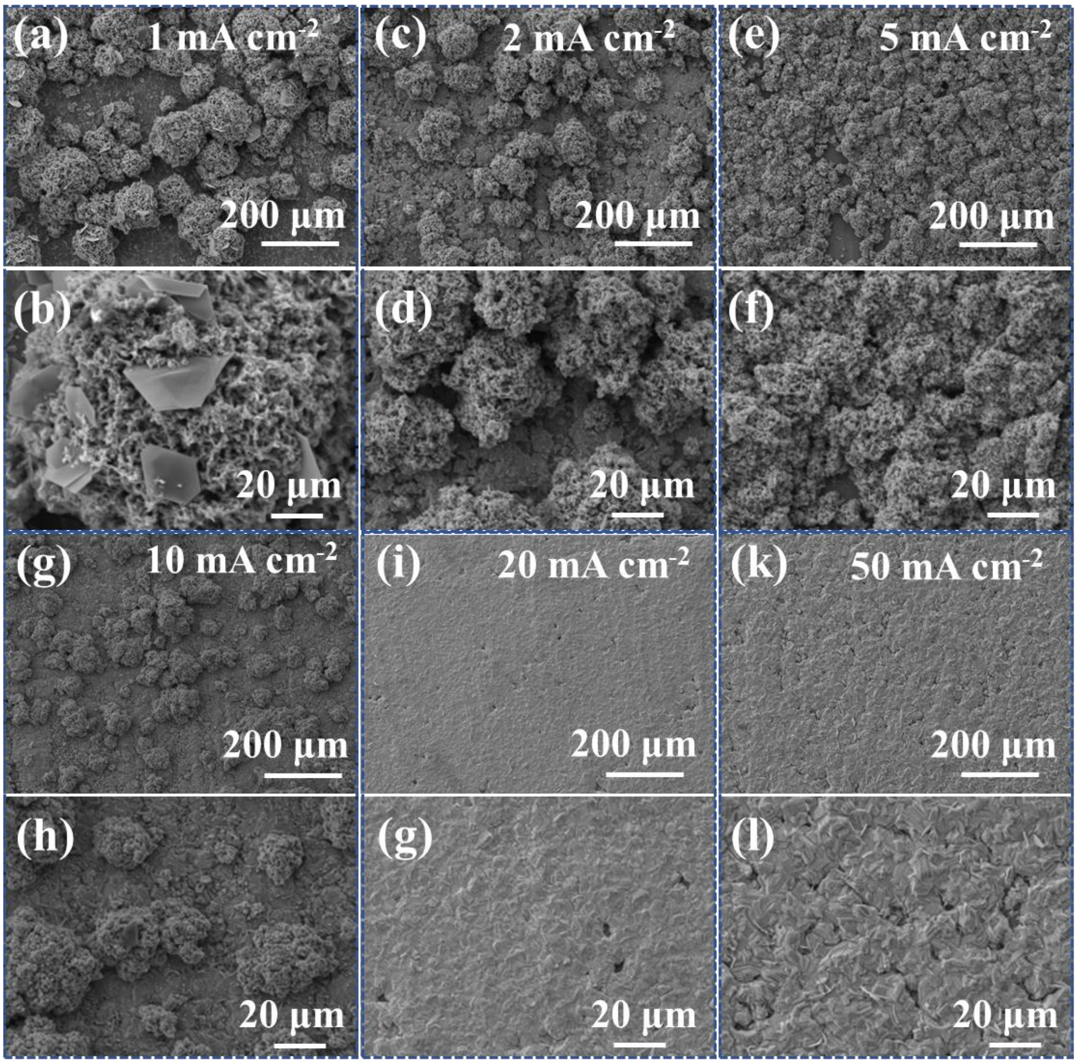

Supplementary Figure 12. The SEM images of Zn morphology deposited on a stainless steel (SS) substrate at different current densities: a,b. 1, c,d. 2, e,f. 5, g,h. 10, i,j. 20, k,l. 50 mA cm^{-2}. The deposition process was conducted in glass cell at ambient temperature (25 °C) with 2M $ZnSO_4$ aqueous solution as electrolyte.

When the substrate switches to SS, the correlation between the current density and the Zn nucleation and morphology is similar: the higher current density contributes to compact and closely packed Zn deposits.

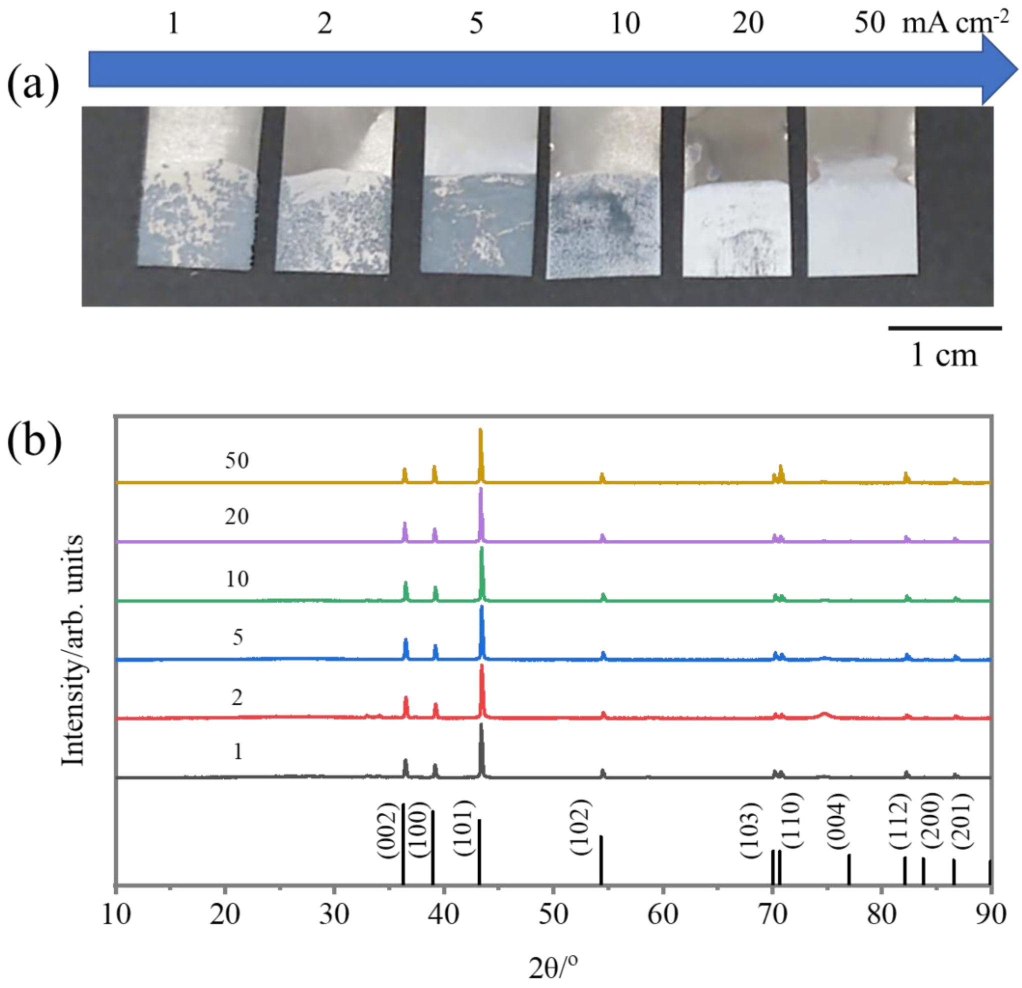

Supplementary Figure 13. a. The photographic picture and b. normalized X-ray diffractograms of the deposited Zn on SS substrate at different current densities. The deposition process was conducted in glass cell at ambient temperature (25 °C) with 2M $ZnSO_4$ aqueous solution as electrolyte.

The color changes of Zn deposits from dark at low current density to grey at high current density, which is similar to the phenomenon witnessed on Cu substrate (Supplementary Figure 13a), indicating the morphology evolution from mossy to

closely packed Zn. X-ray diffractograms support the hypothesis of altered crystallographic texture (Supplementary Figure 13a).

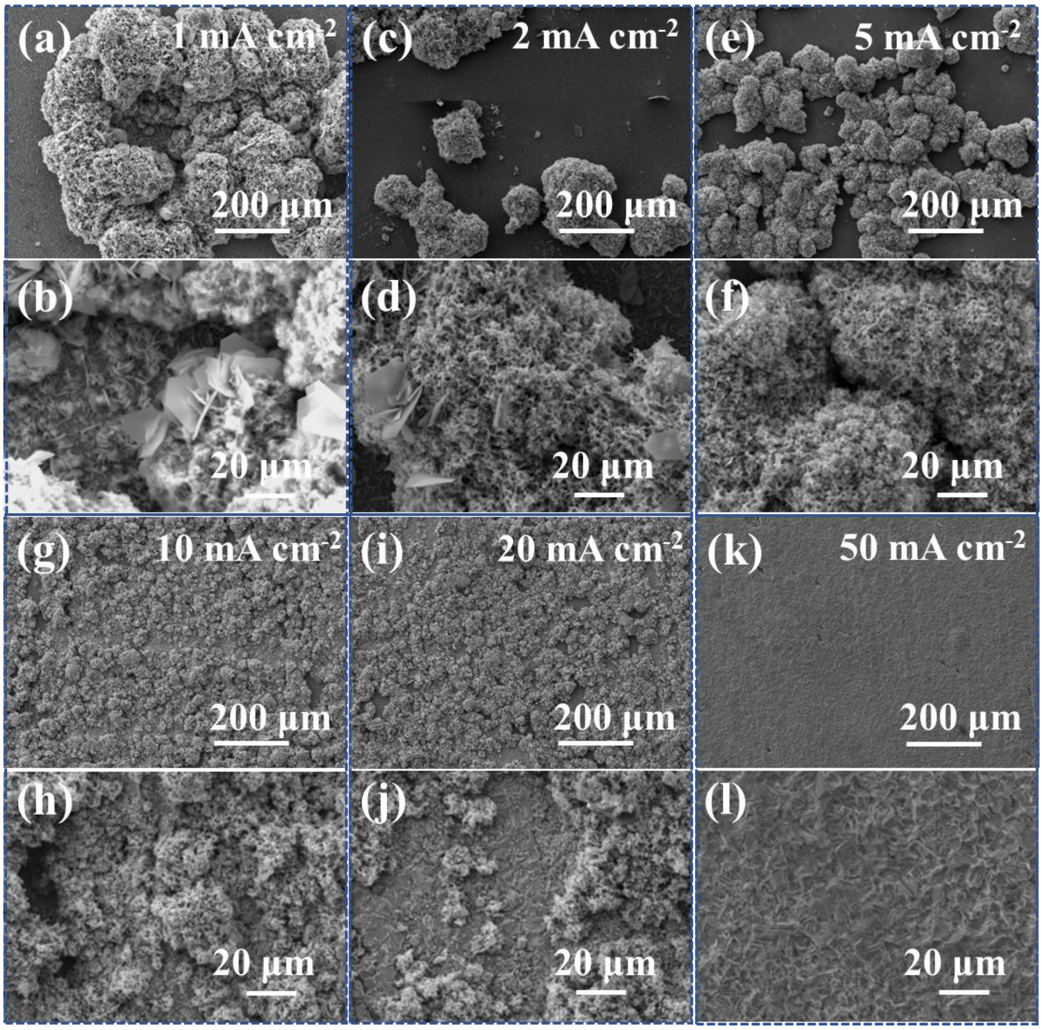

Supplementary Figure 14. The SEM images of Zn morphology deposited on a Ti substrate at different current densities: a,b. 1, c,d. 2, e,f. 5, g,h. 10, i,j. 20, k,l. 50 mA cm^{-2}. The deposition process was conducted in glass cell at ambient temperature (25 °C) with 2M $ZnSO_4$ aqueous electrolyte. The deposition process was conducted in glass cell at ambient temperature (25 °C) with 2M $ZnSO_4$ aqueous electrolyte.

Ti substrate does not affect the relationship between nucleation and current densities applied. The nuclei size (above 200 μm) of Zn deposited on Ti substrate at low current densities of 1, 2 mA cm^{-2} is larger than that of Zn deposited on Cu and SS. While closely-packed and uniform Zn is observed at high current densities[2].

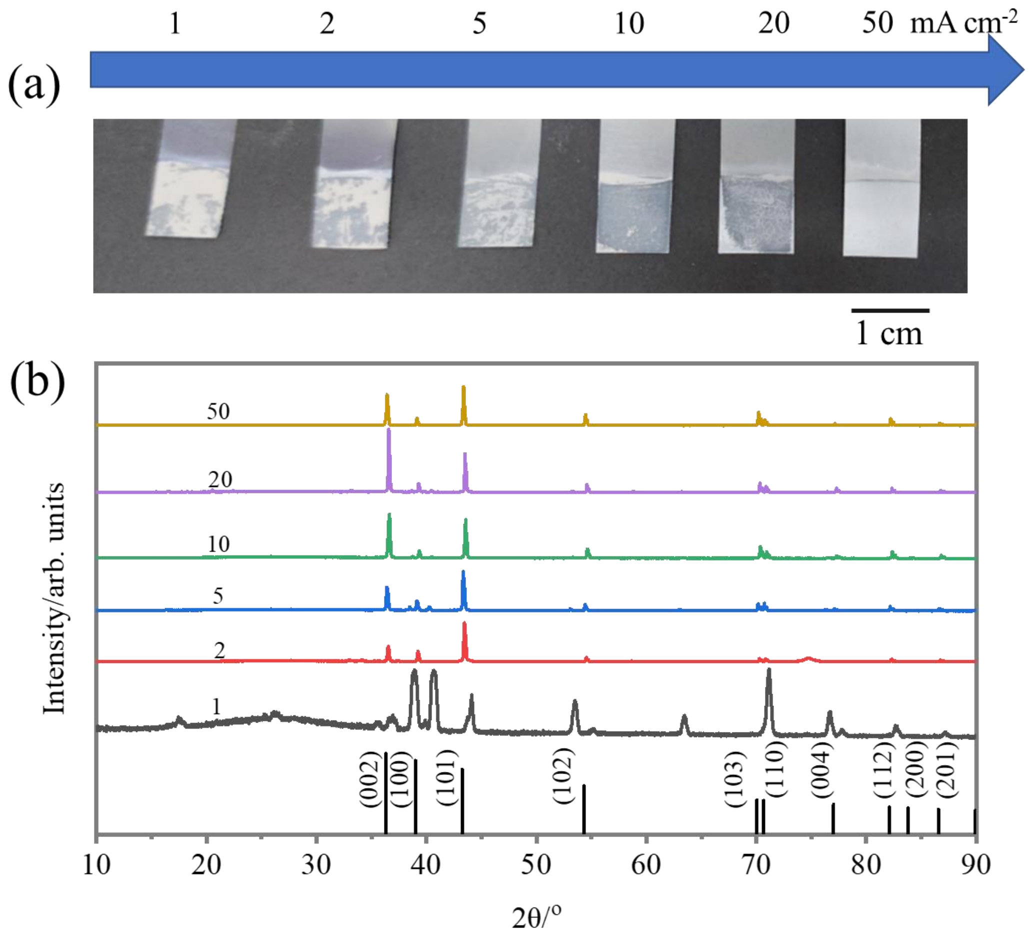

Supplementary Figure 15. a. The photographic picture and b. normalized X-ray diffractograms of the deposited Zn on Ti substrate at different current densities. The deposition process was conducted in glass cell at ambient temperature (25 °C) with 2M $ZnSO_4$ aqueous solution as electrolyte.

The color variation of photographic picture and X-ray diffractograms (Supplementary Figure 15) are in accord with the finding in Supplementary Figure 11,13. The overall trend is similar for different substrates applied. At the same time, some differences can be found for the deposited Zn on Cu, SS, and Ti regarding homogeneity, crystallographic evolution, and optimal current density. This may be attributed to their diverse properties and affinity with Zn.

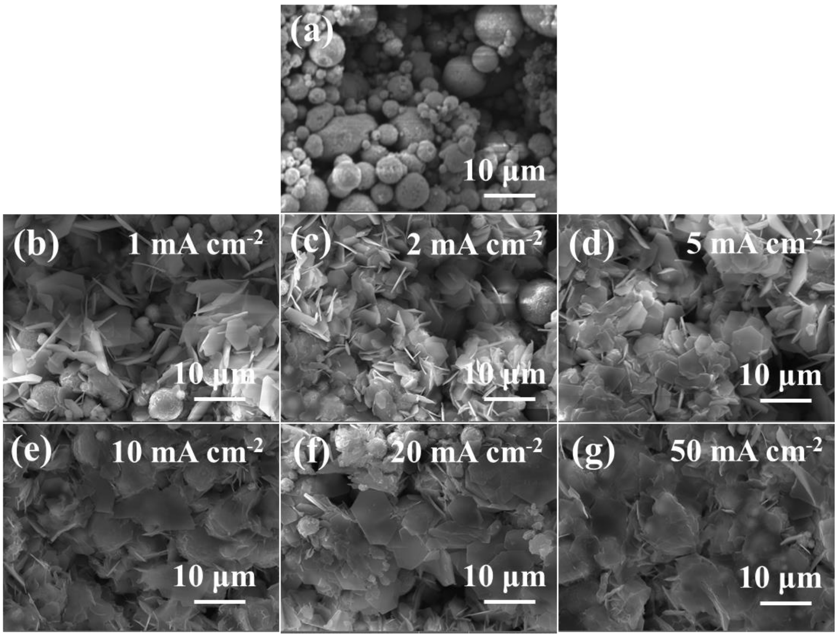

Supplementary Figure 16. The SEM images of Zn powder-based electrodes morphology. a. The initial Zn powder electrode, the Zn deposition morphology at different current densities: a,b. 1, c,d. 2, e,f. 5, g,h. 10, i,j. 20, k,l. 50 mA cm^{-2}. The deposition process was conducted in coin cell at ambient temperature (25 °C) with 2M $ZnSO_4$ aqueous solution as electrolyte.

The difference between Zn foil anode and Zn powder anode is that the deposition substrate is not Zn foil but the residual conductive Zn powers. The deposition morphology of Zn deposits in is also affected by the current density. As the current density increases, the deposited Zn tends to be more compact and uniform (Supplementary Figure 16e,f,g). The Zn powders slightly alter the Zn deposition but do not change the correlation between the current density and the Zn deposition.

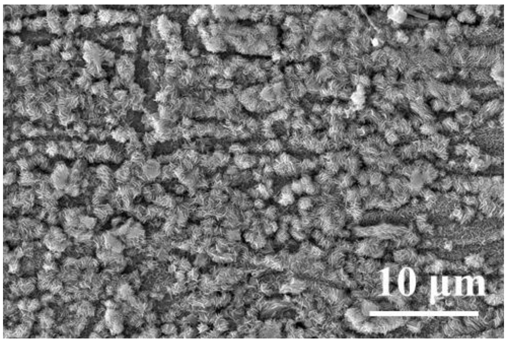

Supplementary Figure 17. The SEM images of PD-Zn created at the current density

of 20 mA cm^{-2}. The deposition process was conducted in coin cell at ambient temperature (25 °C) with 2M $ZnSO_4$ aqueous electrolyte.

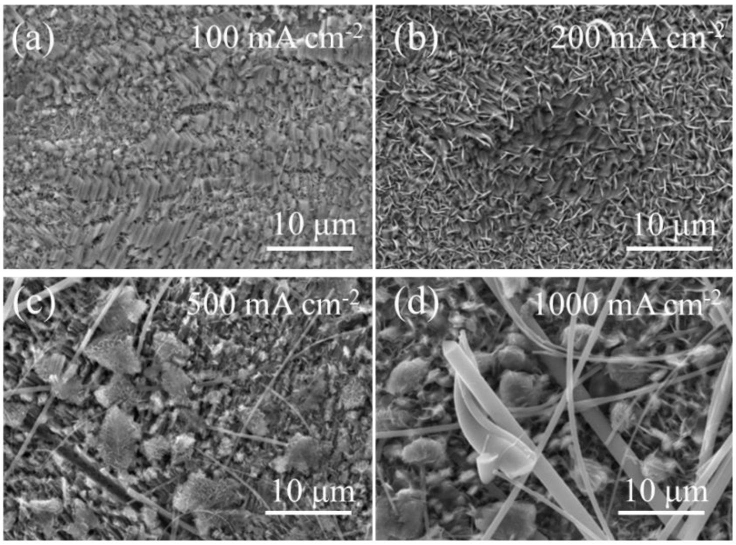

Supplementary Figure 18. The SEM images of PD-Zn created at the current density of a. 100 , b. 200 , c. 500, d. 1000 mA cm^{-2}, respectively. The deposition process was conducted in coin cell at ambient temperature (25 °C) with 2M $ZnSO_4$ aqueous electrolyte.

With further increasing the current density to above 50 mA cm^{-2} and keeping the same capacity (0.3 mAh cm^{-2}), the pre-deposited layer obtained at 100 mA cm^{-2} shows an acceptable uniformity without the dendritic morphology. This layer shows higher heterogeneity and more prominent Zn protrusions when the current density increases to 200, 500, and 1000 mA cm^{-2}. This reason is the ion diffusion becomes the rate-determining step when a high current density is applied. The diffusion restriction leads to the formation of the ion depletion layer near the electrode, incurs dendrites formation, and exaggerates deposition heterogeneity[3].

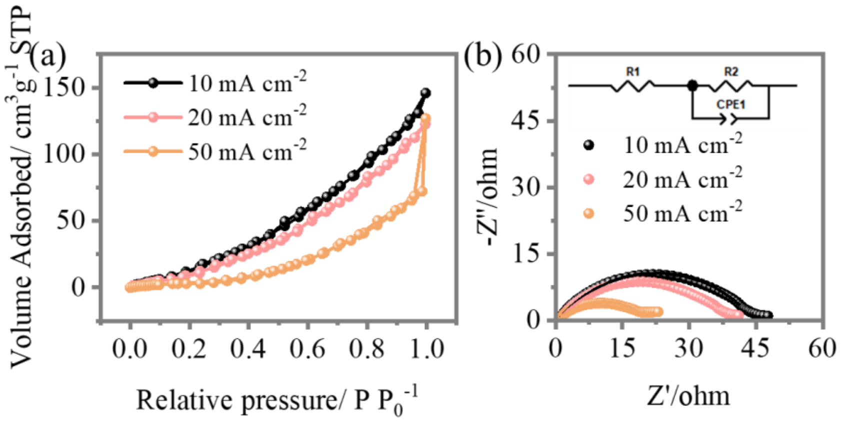

Supplementary Figure 19. a. N_2 adsorption-desorption isotherms of the deposited 10 mAh cm^{-2} of Zn on Cu foil under different current densities. b. The impedance of the PD-Zn||PD-Zn with different pre-deposition current densities.

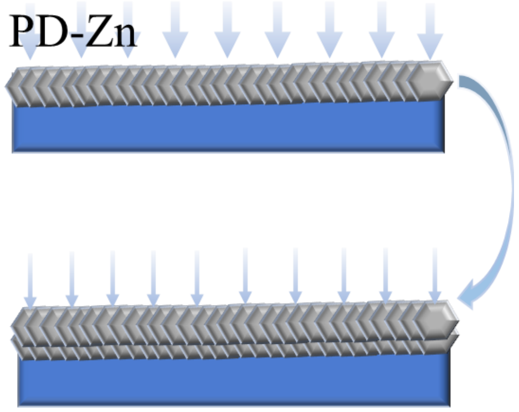

Supplementary Figure 20. The schematic of uniform deposition with the existence of a pre-deposited Zn layer.

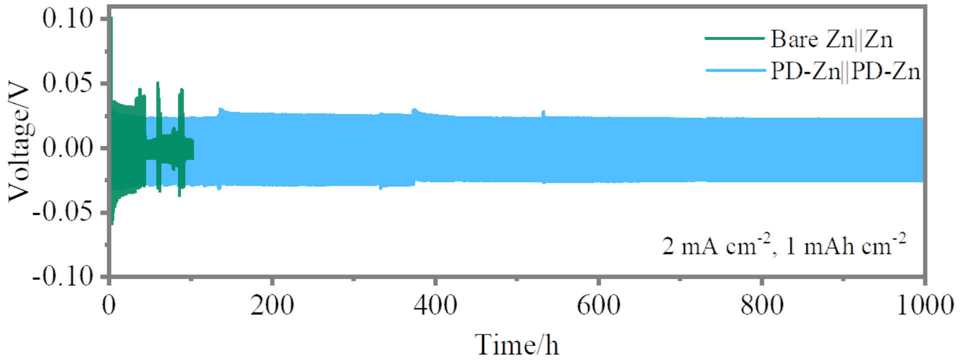

Supplementary Figure 21. The voltage-time profile of Bare Zn||Zn and PD-Zn||PD-Zn symmetric cells cycled at of 2 mA cm^{-2}, 1 mAh cm^{-2}.

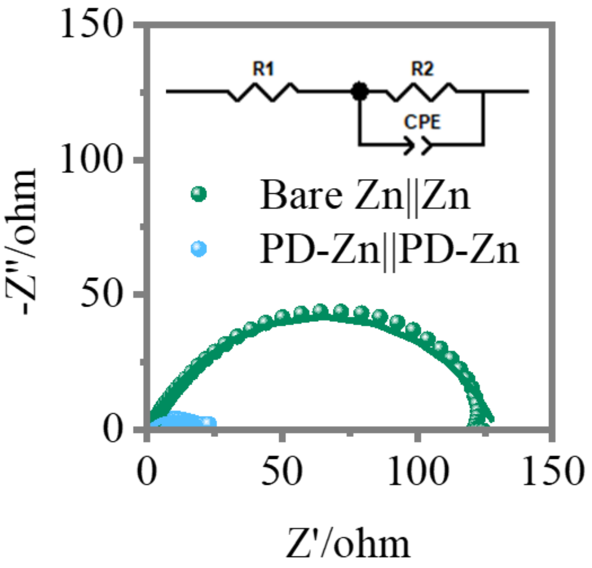

Supplementary Figure 22. The experimented and fitted EIS curve of Bare Zn||Zn and PD-Zn||PD-Zn symmetric cell.

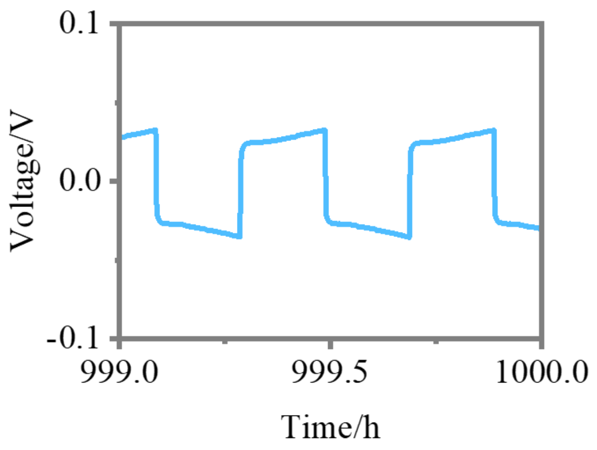

Supplementary Figure 23. The detailed voltage-time profile for PD-Zn||PD-Zn cell at the later stage of cycling with the current density of 2 mA cm^{-2}.

The voltage profile after 1000 h still presents a stable curve similar to the initial cycling, suggesting the stable state of the PD-Zn electrode even after 1000 h.

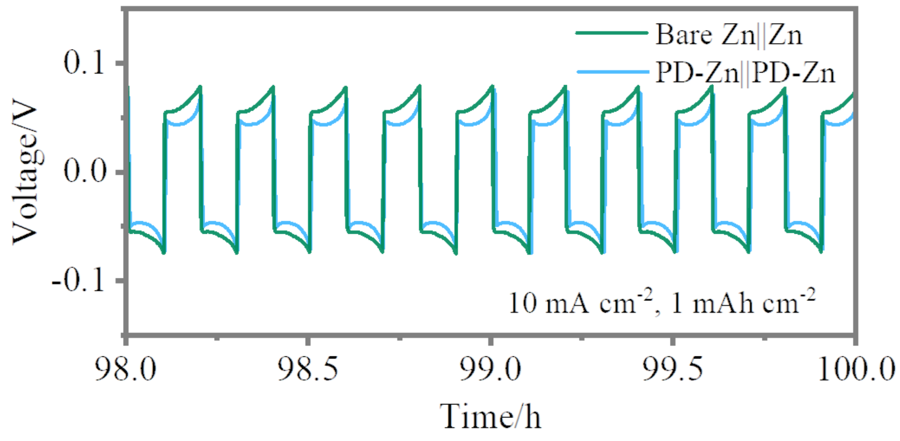

Supplementary Figure 24. The detailed voltage-time profile for PD-Zn||PD-Zn cell cycled at the current density of 10 mA cm^{-2}.

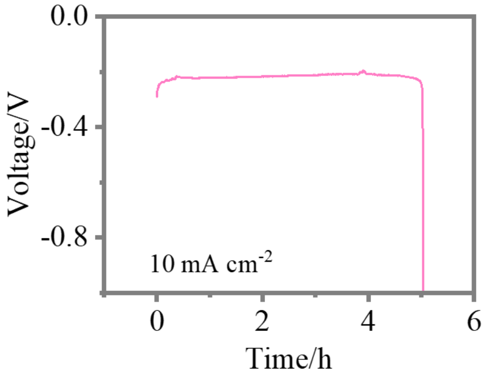

Supplementary Figure 25. Evolution of bare Zn||Zn cell voltage upon plating Zn at a constant current of 10 mA cm^{-2}.

The dramatic voltage decrease implies the characteristic of ion depletion near the electrode[4]. As shown in Supplementary Figure 25, when the current density of 10 mA cm^{-2} is applied, the overpotential starts to dramatically increase after around 5 h (overall plating amount: around 50 mAh cm^{-2}). This is much higher than the reported Li anode with less than 1 h even under the lower current densities[5]. The results verified our hypothesis that the ion depletion time is much longer in the aqueous electrolyte-based Zn anode. This is one reason that the Zn deposits is more densely compact and uniform when cycled at high current densities. Therefore, the cycle life of the bare Zn is also increasing with the current density, and the performance difference between the PD-Zn and bare Zn cycled at high current density is not as obvious as the that cycled at low current density.

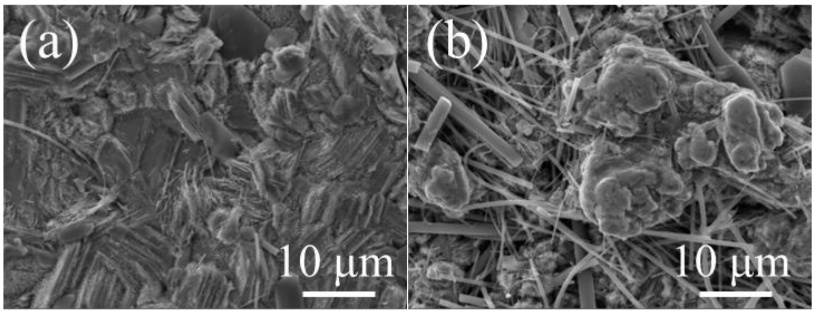

Supplementary Figure 26. The SEM images of the a. PD-Zn and b. bare Zn after 100 cycles at 10 mA cm^{-2}, 1 mAh cm^{-2}. The cycling was conducted in coin cell at ambient temperature (25 °C) with 2M $ZnSO_4$ aqueous electrolyte.

The difference of the PD-Zn and bare Zn cycled at 10 mA cm^{-2} is not as large as the electrodes cycled at 5 mA cm^{-2}, and this can be ascribed to the closely packed crystallographic texture formed at higher current density, which reduced the heterogeneity.

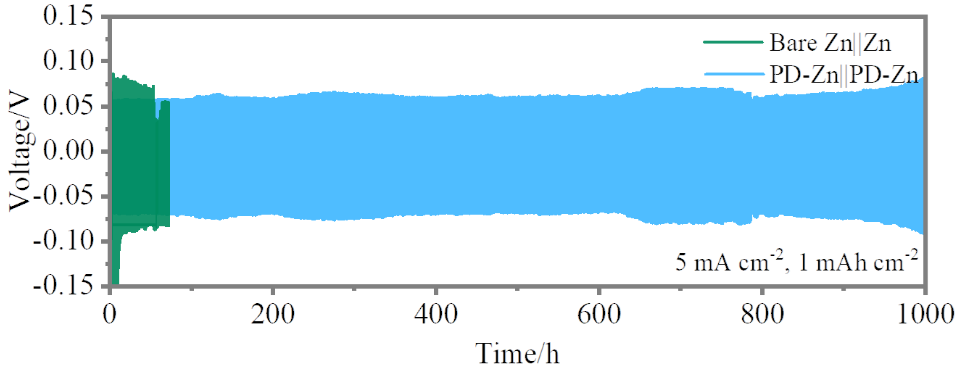

Supplementary Figure 27. The voltage-time profile of Bare Zn||Zn and PD-Zn||PD-Zn symmetric cells with non-woven paper as the separator.

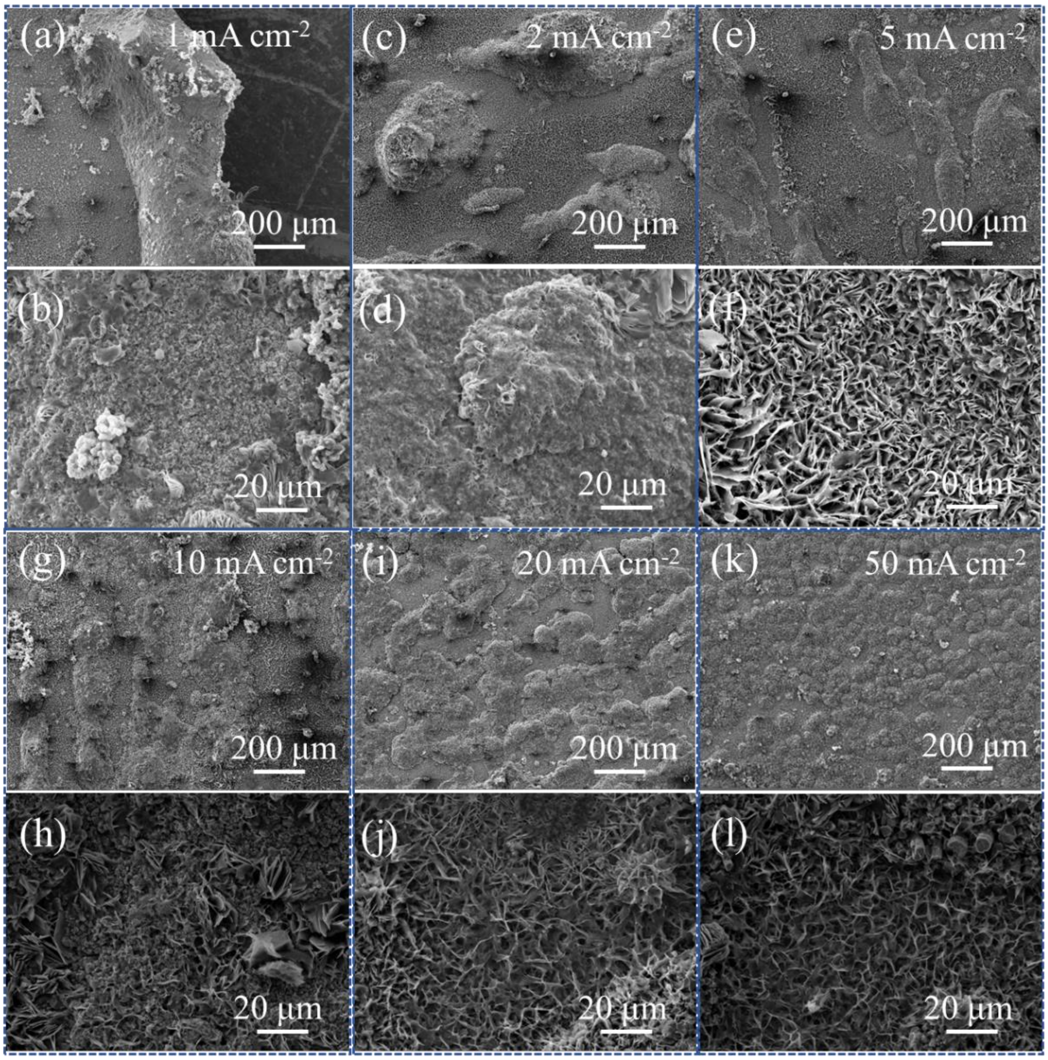

Supplementary Figure 28. The SEM images of Zn morphology deposited on a Cu substrate at different current densities: a,b. 1, c,d. 2, e,f. 5, g,h. 10, i,j. 20, k,l. 50 mA cm^{-2}. The deposition process was conducted in glass cell at ambient temperature (25 °C) with 1M zinc bis(trifluoromethylsulfonyl)imide ($Zn(TFSI)_2$)/H_2O aqueous solution as the electrolyte.

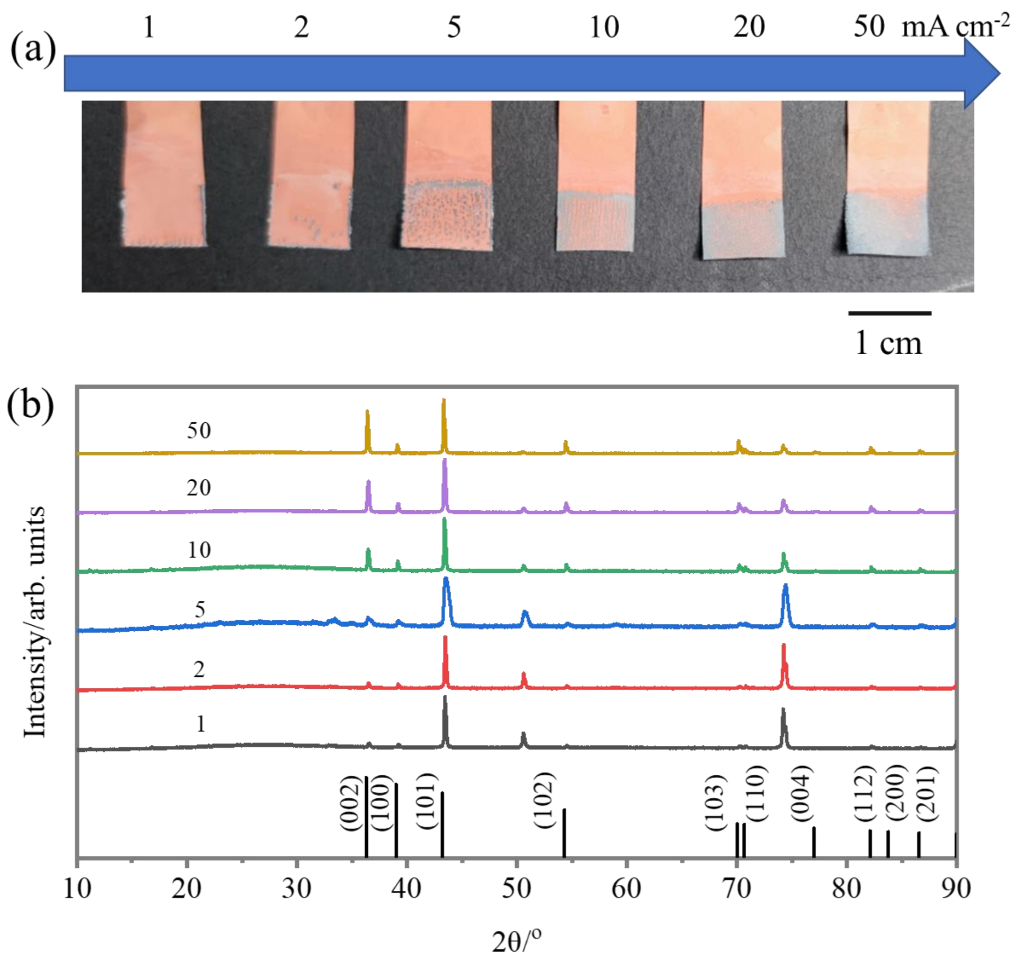

Supplementary Figure 29. a. The photographic picture and b. normalized X-ray diffractograms of the deposited Zn on Cu substrate at different current densities with 1M $Zn(TFSI)_2$ aqueous solution as the electrolyte.

The photographic picture and normalized X-ray diffractograms confirm that the higher uniformity, and altered crystallographic texture under higher current density, which is consistent with the finding in Supplementary Figure 27.

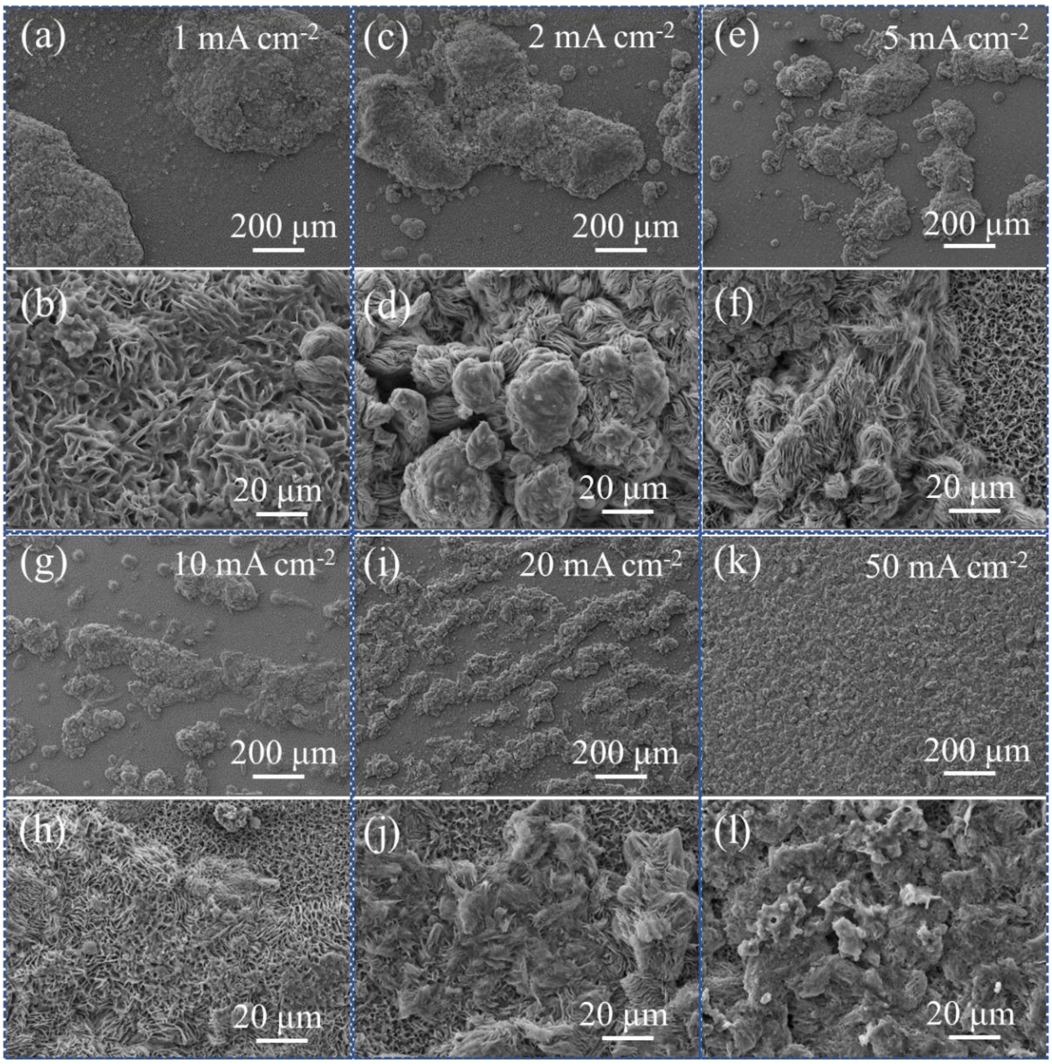

Supplementary Figure 30. The SEM images of Zn morphology deposited on a Cu substrate at different current densities a,b. 1, c,d. 2, e,f. 5, g,h. 10, i,j. 20, k,l. 50 mA cm^{-2}. The deposition process was conducted in glass cell at ambient temperature (25 ℃) with 1M $Zn(TFSI)_2/H_2O$: Dimethoxyethane (DME) (1:1 by volume) as the electrolyte.

The overall trend of Zn uniformity remains no change after adjusting the solvent. The micromorphology is altered which may be attributed to formation of the solid electrolyte interphase formed with the existence of the organic solvent[6].

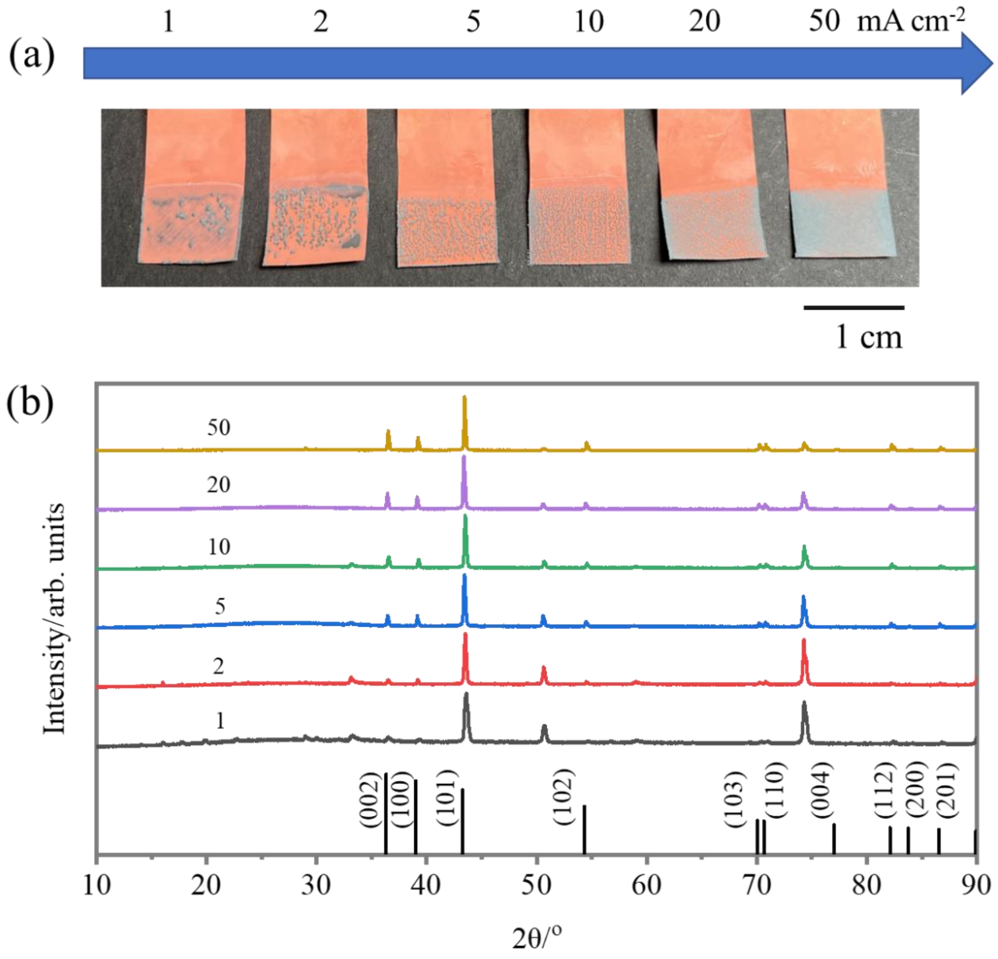

Supplementary Figure 31. a. The photographic picture and b. normalized X-ray diffractograms of the deposited Zn on Cu substrate at different current densities with 1M $Zn(TFSI)_2/H_2O$: DME (1:1 by volume) as the electrolyte.

Compared with the photographic picture in Supplementary Figure 29, the homogeneity demonstrated in Supplementary Figure 31a is much enhanced with the addition of DME as co-solvent, especially at the low current densities (the two specimens on the left). Other phenomena are similar, following the same trend with the increase of current density (Supplementary Figure 31b).

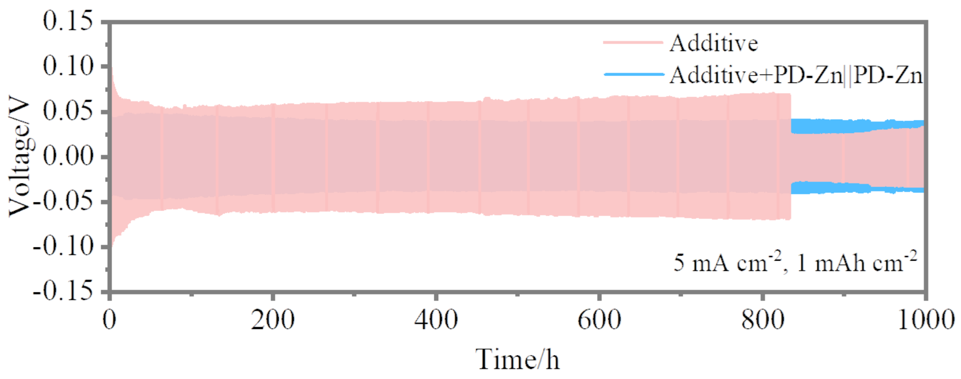

Supplementary Figure 32. The voltage-time profile of bare Zn||Zn and PD-Zn||PD-Zn symmetric cells with the addition of 0.05 M KPF_6 in 2M ZnSO4 aqueous electrolyte.

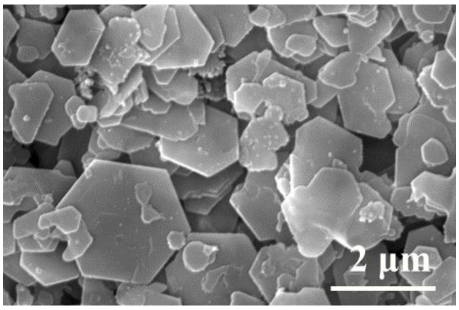

Supplementary Figure 33. The SEM image of the δ-MnO_2 cathode before electrode preparation. The synthesized δ-MnO_2 is lamellar with size ranging from 1 to 3 µm.

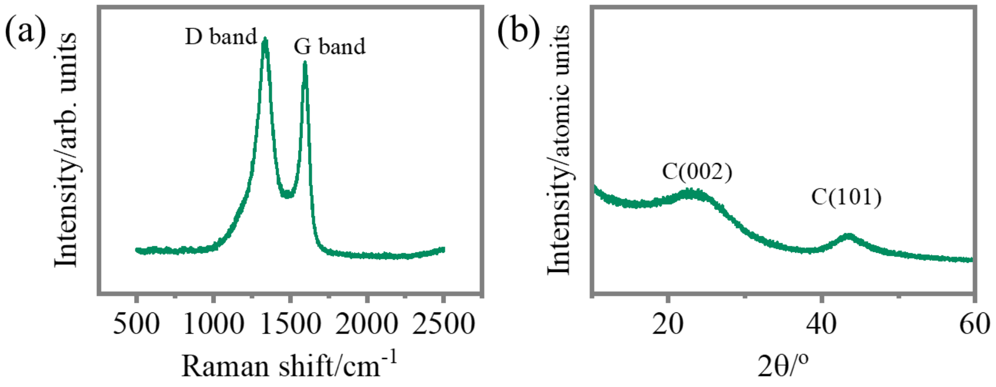

Supplementary Figure 34. a. The Raman spectra and b. X-ray diffractogram of the active carbon.

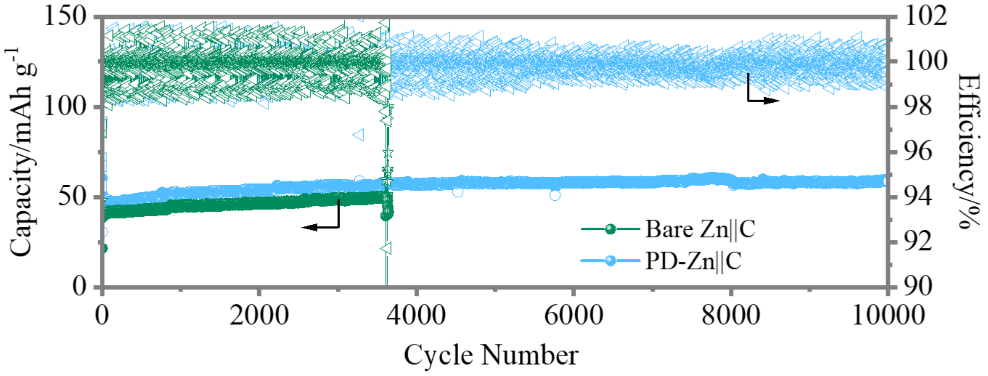

Supplementary Figure 35. long-term cycling performance of PD-Zn||C and bare Zn||C cells.

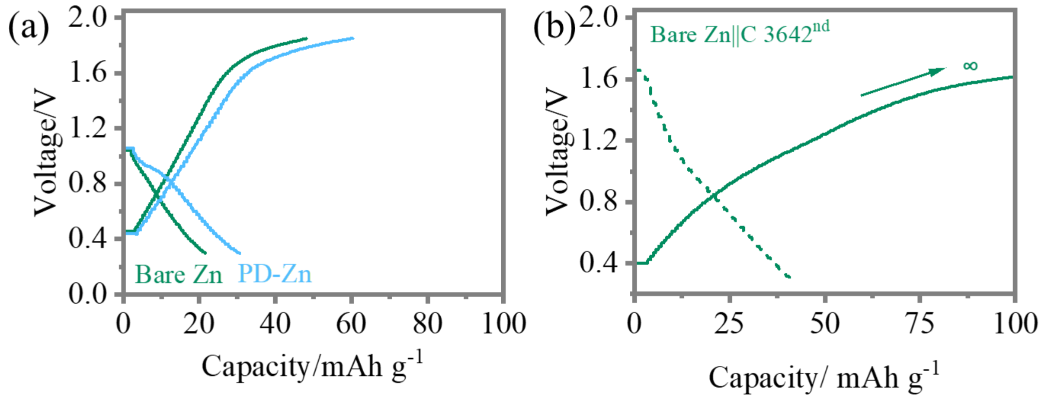

Supplementary Figure 36. a. The initial GCD curves of PD-Zn||C and bare Zn||C cell; b. The 3642nd GCD curve of bare Zn||C.

PD-Zn||C cell demonstrated higher capacity and lower polarization in the GCD curve of the 1st cycle (Supplementary Figure 35a). The charge profile at the end of life (Bare Zn||C cell) shows an infinite capacity which indicates that the short circuit happens.

Supplementary Table 1. The surface area summary of the deposited 10 mAh cm^{-2} of Zn on Cu foil at different current densities.

Current density (mA cm^{-2})	specific surface area (m^2 g^{-1})	Error (%)
10	64.5	1

20	53.8	1
50	13.8	1

Supplementary Table 2. The fitted impendence parameters of PD-Zn symmetric cells with PD-Zn electrode fabricated at different current densities.

Parameters (Ω)	PD-Zn||PD-Zn (10 mA cm^{-2})		PD-Zn||PD-Zn (20 mA cm^{-2})		PD-Zn||PD-Zn (50 mA cm^{-2})	
	Value	Error (%)	Value	Error (%)	Value	Error (%)
R_1	0.67	7.38	0.76	7.6	0.58	5.34
R_2	44.0	2.91	39.35	2.56	20.36	2.92

Supplementary Table 3. The fitted impendence parameters of Bare Zn||Zn and PD-Zn||PD-Zn cells.

Parameters	Bare Zn||Zn		PD-Zn||PD-Zn	
(Ω)	Value	Error (%)	Value	Error (%)
R_1	2.60	1.51	0.58	5.34
R_2	127.9	2.24	20.36	2.92

Supplementary Table 4. Comparison in symmetric cell performance of this work and the recent publications.

Modification	Current density /Areal capacity	Average Overpotential (mV)	Lifespan (h)	Temperature (°C)	Cell type	Electrolyte volume (μL)	Thickness of electrode (μm)	Lateral size of electrode	Ref
ZnO@Zn	5 mA cm^{-2} 1.25 mAh cm^{-2}	43	500	NA	NA	NA	100	Diameter： 15 mm	8
PVB@Zn	0.5 mA cm^{-2} 0.5 mAh cm^{-2}	42.1	2200	25	Coin cell	NA	NA	NA	9
Zn@Cu foam with PAM additive	2 mA cm^{-2} 4 mAh cm^{-2}	63.5	280	NA	Coin cell	NA	NA	NA	10
ZnP@Zn	10 mA cm^{-2} 2.5 mAh cm^{-2}	90	1900	NA	Coin cell	NA	10	NA	11
(002) Zn foil	1 mA cm^{-2} 1 mAh cm^{-2}	71	500	NA	Coin cell	NA	500	NA	12
TiO2@Zn	1 mA cm^{-2} 1 mAh cm^{-2}	30	460	NA	Coin cell	NA	NA	NA	13
Zn@Cu foam	2 mA cm^{-2} 1 mAh cm^{-2}	40	150	NA	Coin cell	NA	NA	NA	14
Kaolintu@Zn	4.4 mA cm^{-2} 1.1 mAh cm^{-2}	70	800	NA	Coin cell	NA	100	Diameter： 15 mm	15
ZnF2@Zn	0.5 mA cm^{-2} 0.5 mAh cm^{-2}	60	800	NA	NA	NA	30	Diameter： 12 mm	16
Zn88Al12	0.5 mA cm^{-2} 0.5 mAh cm^{-2}	30	2000	NA	NA	NA	30	0.5 cm x 0.5 cm	17
ZnS@Zn	2 mA cm^{-2} 2 mAh cm^{-2}	50	1100	25	Coin cell	NA	10	NA	18

Zn-based montmorillonite@Zn	1 mA cm^{-2} 0.25 mAh cm^{-2}	50	1000	NA	Coin cell	40	100	Diameter：12 mm	19
3D ZnF2@Zn	1 mA cm^{-2} 1 mAh cm^{-2}	35	800	NA	Coin cell	NA	100	NA	20
Zn (SN@Glass fiber seperator)	5 mA cm^{-2} 5 mAh cm^{-2}	70	1000	NA	Coin cell	NA	NA	NA	21
Deposited (002) Zn	1 mA cm^{-2} 0.2 mAh cm^{-2}	49	800	NA	Coin cell	NA	50	NA	22
PD-Zn	5 mA cm^{-2} 1 mAh cm^{-2}	32	1000	25±1	Coin cell	100	80	1x1 cm	This work

Supplementary Table 5. The fitted impendence parameters of Zn||MnO_2 cells.

Parameters	Bare Zn||MnO_2		PD-Zn||MnO_2	
(Ω)	Value	Error (%)	Value	Error (%)
R_1	2.089	2.34	3.22	3.18
R_2	66.66	2.29	47.08	2.89
Z_w	270.4	4.97	0.64	5.51

Supplementary Table 6. The Comparison in Zn||MnO_2 cell performance of this work and the recent publications.

Cathode	Anode	Electrolyte	Capacity (mAh g^{-1})	Temperature	Cell type	Electrolyte amount (μL)	Cathode percentage (wt%)	Loading（mg cm-2）	Thickness of cathode electrode (μm)	Lateral size of cathode electrode	Thickness of Zn anode	Lateral size of anode	Capacity retention	Ref
α-MnO_2/CNT	Zn	zinc alginate gel (2M $ZnSO_4$+0.2M $MnSO_4$)	250	NA	Coin cell	NA	NA	NA	NA	NA	NA	NA	300 after 130 cycles at 0.2 A g^{-1}	23
Electrodeposited MnO_2	poly (vinyl butyral) coating	1M $ZnSO_4$+0.1M $MnSO_4$	135	25	Coin cell	NA	100	1.12	NA	NA	NA	NA	130 after 1500 cycles at 1.54 A g^{-1}	9

Commercial MnO_2	Zn@3D Cu foam	1 M $ZnSO_4$, 0.5 M Na_2SO_4, 1 g L^{-1} PAM, 0.1 M $MnSO_4$	95	25	Coin cell	NA	NA	NA	NA	NA	10	NA	92 after 600 cycles at 1 A g^{-1}	10
CNT/MnO_2	ZnP@Zn	2 M $ZnSO_4$ + 0.1 m $MnSO_4$	180	NA	Coin cell	NA	70	NA	NA	Diameter: 10 mm	500	NA	154 after 1000 cycles at 1 A g^{-1}	11
MnO_2	(002) Zn foil	2 M $ZnSO_4$ + 0.1 m $MnSO_4$	120	NA	Coin cell	NA	70	1-1.5	NA	NA	NA	NA	1800 cycles after 1800 at 0.5 A g^{-1}	12
MnO_2	Zn (SN@Glass fiber seperator)	2 M $ZnSO_4$ + 0.2 M $MnSO_4$,	170	NA	Coin cell	NA	70	NA	NA	NA	NA	NA	200 after 600 cycles at 0.3 A g-1	21
MnO_2	Zn (Fluorinated COF separator)	NA	130	NA	Coin cell	NA	70	8	NA	NA	NA	NA	120 after 1000 cycles at 1.54 A g^{-1}	24
σ-MnO_2	Zn	0.5 M $ZnSO_4$ + 0.1 M $MnSO_4$+0.5 M triethylmethyl-ammonium sulfate	150	NA	Coin cell	NA	80	1.6	NA	NA	80	NA	120 after 680 cycles at 0.5 A g^{-1}	25
MnO_2	Zn	0.5 M	210	NA	Coin	NA	80	1-2	NA	Diameter:	20	NA	190 after 200	26

		$ZnSO_4$+0.5 M triethylamine sulfate			cell					12 mm			cycles at 0.2 A g^{-1}	
Commercial MnO_2	Zn@TiO_2	1 M $ZnSO_4$ + 0.1 M MnSO4	100	NA	Coin cell	NA	NA	NA	NA	NA	NA	NA	80 after 300 cycles at 1 A g^{-1}	13
β-MnO_2	Cu foam@Zn	2 M $ZnSO_4$ + 0.1 m $MnSO_4$	150	NA	Coin cell	NA	NA	NA	NA	NA	NA	NA	210 after 500 cycles at 1 A g^{-1}	14
Commercial MnO_2	Zn (GF@GO separator)	2 M $ZnSO_4$ + 0.5 m $MnSO_4$	82	NA	Coin cell	NA	70	NA	NA	NA	NA	NA	62 after 500 cycles at 0.5 A g^{-1}	27
α-MnO_2	3D zinc anodes	2 M $ZnSO_4$ + + 0.1 M $MnSO_4$ + 0.05 mM TBA2SO_4	220	NA	Coin cell	NA	70	1	20	NA	3	NA	210 after 300 cycles at 1 A g^{-1}	28
MnO_2/graphite	Zn (cellulose/ GO separator)	2 M $ZnSO_4$ + 0.5 M $MnSO_4$	80	NA	Coin cell	NA	80	1.3-1.6	NA	NA	NA	NA	70 after 1000 cycles at 1 A g^{-1}	29
MnO_2	Zn with CNF interlayer	2 M $ZnSO_4$+0.2 M $MnSO_4$	200	NA	Coin cell	NA	70	7.23	NA	NA	NA	NA	215 after 400 cycles at 1 A g^{-1}	30
δ-MnO_2	PD-Zn	2 M $ZnSO_4$+0.1 M MnSO4	180	25±1° C	Coin cell	100	70	5.6	1770	1×1 cm	80	1×1 cm	90 after 2000 cycles at 1 A g^{-1}	This work

Supplementary Note 1. Specific Surface Area of the electrodeposited Zn on Cu

For the surface area, we conducted a supplementary experiment. 10 mAh cm^{-2} amount of Zn was deposited on Cu foil (1×1 cm) with different current densities (10, 20, 50 mAh cm^{-2}), and surface areas were measured. Based on the BET measurement (multipoint BET method) derived from Supplementary Figure 19a, the BET surface area data are summarized in Supplementary Table 1. The surface area is inversely proportional to the current density applied, and the surface area of Zn under 50 mAh cm^{-2} is only 13.8 m^2 g^{-1}, which is less than a quarter of the number (64.5 m^2 g^{-1}) of Zn deposited under 10 mAh cm^{-2}. This is well in accord with the SEM images in Supplementary Figure 10, in which the deposited Zn under the higher current density is denser and more compact. A pre-deposited Zn layer in Zn foil will lead to a higher surface area than that of bare Zn, but the surface area decreases with the current density increase.

Supplementary Note 2. EIS of symmetric Zn||Zn cells

The impedance of symmetric cells with different pre-deposition current densities (10, 20, 50 mA cm^{-2}) was also measured, and the results (Supplementary Figure 19b, Supplementary Table 2) show that the charge transfer resistance (R_{ct}) is much lower with high pre-deposition current (only less than 20 Ω with the current density of 50 mA cm^{-2}). R_{ct} is associated with the surface area, temperature, desolvation energy and interfacial chemistry, and so, on. In this study, considering the electrolyte and temperature is the same, and less surface area but lower impedance with high pre-deposition current indicates that the surface area and local current density is not the only working mechanism in this pre-deposition strategy.

Supplementary Notes 3. The influence of the thickness separator

The thickness of the separator is highly related to the cell lifespan. As shown in Supplementary Figure 27, the thinner non-woven separator (113 μm) is indeed more sensitive to short-circuit, and the bare Zn||Zn cell with non-woven paper separator demonstrates much less lifespan compared with their counterpart with a glass fiber separator (Figure 4d). However, the PD-Zn||PD-Zn cell can still achieve a better life span (more than 1000 h) and lower overpotential compared with those of the bare Zn

cells.

Supplementary Notes 4. The influence of electrolyte

The electrolyte plays a vital role in determining the Zn morphology. To investigate the effect of electrolytes, the electrolyte is switched from 2M $ZnSO_4$ to 1M $Zn(TFSI)_2$ aqueous solution and 1M $Zn(TFSI)_2/H_2O$: Dimethoxyethane (DME) (1:1 by volume). Overall, the nuclei density and uniformity in those electrolytes follow a similar trend with 2M $ZnSO_4$ electrolyte that the higher current density leads to a higher nuclei density and more uniform Zn deposition (Supplementary Figure 28-31).

Supplementary Notes 5. The influence of electrolyte additive

To validate the effect of the pre-deposition strategy on the base of the electrolyte modification, we added 0.05 M KPF_6 into the 2M $ZnSO_4$ electrolyte, which has been proven to stabilize the Zn electrode interface[7]. As the cycling performance demonstrated in Supplementary Figure 31, the cell's lifespan has also been greatly enhanced (over 1000 h) relative to the pure electrolyte modification (around 800 h).

Reference list

1. Sun KEK, Hoang TKA, Doan TNL, Yu Y, Chen P. Highly Sustainable Zinc Anodes for a Rechargeable Hybrid Aqueous Battery. *Chemistry* **24**, 1667-1673 (2018).
2. Zhang Y, Howe JD, Ben-Yoseph S, Wu Y, Liu N. Unveiling the Origin of Alloy-Seeded and Nondendritic Growth of Zn for Rechargeable Aqueous Zn Batteries. *ACS Energy Lett.* **6**, 404-412 (2021).
3. Xu X*, et al.* Role of Li-Ion Depletion on Electrode Surface: Underlying Mechanism for Electrodeposition Behavior of Lithium Metal Anode. *Adv. Energy Mater.* **10**, 2002390 (2020).
4. Pang Q, Liang X, Shyamsunder A, Nazar LF. An In Vivo Formed Solid Electrolyte Surface Layer Enables Stable Plating of Li Metal. *Joule* **1**, 871-886 (2017).
5. Bai P, Li J, Brushett FR, Bazant MZ. Transition of lithium growth mechanisms in liquid electrolytes. *Energy Environ. Sci.* **9**, 3221-3229 (2016).
6. Cui J*, et al.* Improved electrochemical reversibility of Zn plating/stripping: a promising approach to suppress water-induced issues through the formation of H-bonding. *Mater. Today Energy* **18**, 100563 (2020).
7. Chu Y, Zhang S, Wu S, Hu Z, Cui G, Luo J. In situ built interphase with high interface energy and fast kinetics for high performance Zn metal anodes. *Energy Environ. Sci.* **14**, 3609-3620 (2021).

8. Xie X, *et al.* Manipulating the ion-transfer kinetics and interface stability for high-performance zinc metal anodes. *Energy Environ. Sci.* **13**, 503-510 (2020).
9. Hao J, *et al.* Designing Dendrite-Free Zinc Anodes for Advanced Aqueous Zinc Batteries. *Adv. Funct. Mater.* **30**, 2001263 (2020).
10. Zhang Q, *et al.* The Three-Dimensional Dendrite-Free Zinc Anode on a Copper Mesh with a Zinc-Oriented Polyacrylamide Electrolyte Additive. *Angew. Chem. Int. Ed. Engl.* **58**, 15841-15847 (2019).
11. Cao P, *et al.* Fast-Charging and Ultrahigh-Capacity Zinc Metal Anode for High-Performance Aqueous Zinc-Ion Batteries. *Adv. Funct. Mater.* **31**, 2100398 (2021).
12. Zhou M, *et al.* Surface-Preferred Crystal Plane for a Stable and Reversible Zinc Anode. *Adv. Mater.* **33**, e2100187 (2021).
13. Zhang Q, *et al.* Revealing the role of crystal orientation of protective layers for stable zinc anode. *Nat. Commun.* **11**, 3961 (2020).
14. Li C, *et al.* Spatially homogeneous copper foam as surface dendrite-free host for zinc metal anode. *Chem. Eng. J.* **379**, 122248 (2020).
15. Deng C, *et al.* A Sieve-Functional and Uniform-Porous Kaolin Layer toward Stable Zinc Metal Anode. *Adv. Funct. Mater.* **30**, 2000599 (2020).
16. Han J, *et al.* A Thin and Uniform Fluoride-Based Artificial Interphase for the Zinc Metal Anode Enabling Reversible Zn/MnO2 Batteries. *ACS Energy Lett.* **6**, 3063-3071 (2021).
17. Wang SB, *et al.* Lamella-nanostructured eutectic zinc-aluminum alloys as reversible and dendrite-free anodes for aqueous rechargeable batteries. *Nat. Commun.* **11**, 1634 (2020).
18. Hao J, *et al.* An In-Depth Study of Zn Metal Surface Chemistry for Advanced Aqueous Zn-Ion Batteries. *Adv. Mater.* **32**, e2003021 (2020).
19. Yan H, Li S, Nan Y, Yang S, Li B. Ultrafast Zinc–Ion–Conductor Interface toward High-Rate and Stable Zinc Metal Batteries. *Adv. Energy Mater.* **11**, 2100186 (2021).
20. Yang Y, *et al.* Synergistic Manipulation of Zn(2+) Ion Flux and Desolvation Effect Enabled by Anodic Growth of a 3D ZnF2 Matrix for Long-Lifespan and Dendrite-Free Zn Metal Anodes. *Adv. Mater.* **33**, e2007388 (2021).
21. Hou Z, Gao Y, Tan H, Zhang B. Realizing high-power and high-capacity zinc/sodium metal anodes through interfacial chemistry regulation. *Nat. Commun.* **12**, 3083 (2021).
22. Yuan D, *et al.* Anion Texturing Towards Dendrite-Free Zn Anode for Aqueous Rechargeable Batteries. *Angew. Chem. Int. Ed. Engl.* **60**, 7213-7219 (2021).
23. Tang Y, *et al.* Ion-confinement effect enabled by gel electrolyte for highly reversible dendrite-free zinc metal anode. *Energy Stor. Mater.* **27**, 109-116 (2020).
24. Zhao Z, *et al.* Horizontally arranged zinc platelet electrodeposits modulated by fluorinated covalent organic framework film for high-rate and durable aqueous zinc ion batteries. *Nat. Commun.* **12**, 6606 (2021).
25. Yao R, *et al.* A Versatile Cation Additive Enabled Highly Reversible Zinc Metal

Anode. *Adv. Energy Mater.* **12**, 2102780 (2021).

26. Qian L*, et al.* Cations Coordination-Regulated Reversibility Enhancement for Aqueous Zn-Ion Battery. *Adv. Funct. Mater.* **31**, 2105736 (2021).
27. Cao J, Zhang D, Zhang X, Sawangphruk M, Qin J, Liu R. A universal and facile approach to suppress dendrite formation for a Zn and Li metal anode. *J. Mater. Chem. A* **8**, 9331-9344 (2020).
28. Bayaguud A, Luo X, Fu Y, Zhu C. Cationic Surfactant-Type Electrolyte Additive Enables Three-Dimensional Dendrite-Free Zinc Anode for Stable Zinc-Ion Batteries. *ACS Energy Lett.* **5**, 3012-3020 (2020).
29. Cao J*, et al.* Manipulating Crystallographic Orientation of Zinc Deposition for Dendrite-free Zinc Ion Batteries. *Adv. Energy Mater.* **11**, 2101299 (2021).
30. Liang Y*, et al.* Functionalized carbon nanofiber interlayer towards dendrite-free, Zn-ion batteries. *Chem. Eng. J.* **425**, 131862 (2021).
